# The population genetics of nonmigratory Allen’s Hummingbird (*Selasphorus sasin sedentarius*) following a recent mainland colonization

**DOI:** 10.1002/ece3.7174

**Published:** 2021-01-20

**Authors:** Brian M. Myers, Kevin J. Burns, Christopher J. Clark, Alan Brelsford

**Affiliations:** ^1^ Department of Biology San Diego State University San Diego CA USA; ^2^ Department of Evolution, Ecology, and Organismal Biology Speith Hall University of California Riverside CA USA

**Keywords:** Channel Islands, differentiation, niche, nucleotide diversity, population genetics

## Abstract

Allen's Hummingbird comprises two subspecies, one migratory (*Selasphorus sasin sasin*) and one nonmigratory (*S. s. sedentarius*). The nonmigratory subspecies, previously endemic to the California Channel Islands, apparently colonized the California mainland on the Palos Verdes Peninsula some time before 1970 and now breeds throughout coastal southern California. We sequenced and compared populations of mainland nonmigratory Allen's Hummingbird to Channel Island populations from Santa Catalina, San Clemente, and Santa Cruz Island. We found no evidence of founder effects on the mainland population. Values of nucleotide diversity on the mainland were higher than on the Channel Islands. There were low levels of divergence between the Channel Islands and the mainland, and Santa Cruz Island was the most genetically distinct. Ecological niche models showed that rainfall and temperature variables on the Channel Islands are similar in the Los Angeles basin and predicted continued expansion of nonmigratory Allen's Hummingbird north along the coast and inland. We also reviewed previous genetic studies of vertebrate species found on the Channel Islands and mainland and showed that broad conclusions regarding island–mainland patterns remain elusive. Challenges include the idiosyncratic nature of colonization itself as well as the lack of a comprehensive approach that incorporates similar markers and sampling strategies across taxa, which, within the context of a comparative study of island–mainland relationships, may lead to inconsistent results.

## Graphical abstract

The previously island endemic nonmigratory Allen's Hummingbird colonized the southern California mainland some time before 1970. Here, we compare the founding mainland population in a population genetic context to the southern islands and the northern Santa Cruz Island. We also implement ecological niche modeling to assess whether nonmigratory Allen's Hummingbird, which has rapidly expanded its range across mainland southern California, is projected to continue to expand its range in the near future and possibly encroach upon the range of migratory Allen's Hummingbird.

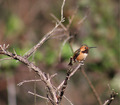

## 1 INTRODUCTION

When island populations colonize the mainland (or vice versa), a founder effect, a type of bottleneck where a founding population descends from a small number of colonizing individuals with limited genetic diversity, could result (Barton & Charlesworth, 1984). For instance, the colonization of the Channel Islands of California by the Golden Eagle (*Aquila chrysaetos*) from the mainland resulted in a founder effect on the islands, where lower genetic diversity was observed (Sonsthagen et al., 2012). In populations with reduced evolutionary potential, such as those with little functional genetic diversity due to the consequences of drift, connectivity can improve genetic diversity and counteract stochastic drift effects, as incoming alleles add genetic diversity to the population via gene flow (Finlay et al., 2017; Hedrick & Kalinowski, 2000; Nei et al., 1975). Thus, investigation of genetic diversity, population connectivity, and potential founder effects shed light on mainland‐island dynamics and the degree of success of colonizing populations (Haig, 1998; Hedrick, 2004).

In addition to population genetic factors, the availability of suitable habitat plays a role in the success of a colonizing population (Braasch et al., 2019; Güthlin et al., 2011). When suitable habitat is sparse, it is difficult to increase initially low levels of genetic diversity, as carrying capacity and the potential for gene flow are limited (Sonsthagen et al., 2012). Therefore, a combination of population genetic factors and availability of suitable habitat influence evolutionary potential and the longevity of colonizing populations.

The California Channel Islands comprise eight primary islands located off the coast of southern California and provide a natural laboratory for studying island–mainland population dynamics. These primary islands form two groups based on their geographic proximity to each other: the southern (San Clemente, San Nicolas, Santa Barbara, and Santa Catalina) and northern (Anacapa, San Miguel, Santa Cruz, Santa Rosa) islands. Although the northern islands were never connected to the mainland, the northern islands formed one large island due to lower sea levels during the late Pleistocene. The northern islands were only about six km from the mainland until temperatures increased, glacial recession occurred, and sea levels rose (Johnson, 1977). The Channel Islands may have also served as a glacial refugium during the Last Glacial Maximum (LGM; Johnson, 1977).

There are 41 species of terrestrial birds that breed on the Channel Islands, including 1 endemic species and 15 endemic subspecies (Johnson, 1972; Schoenherr et al., 2003). Seven of these endemics have been studied and compared to the mainland within a population genetic context (Caballero & Ashley, 2011; Delaney & Wayne, 2005; Hanna et al., 2019; Karin et al., 2018; Mason et al., 2014; Rutledge et al., 2017; Wilson et al., 2015). Because genetic connectivity between the Channel Islands and California has only been studied for some breeding bird species, we lack a general consensus on island–mainland relationships. Colonization events of the mainland from the Channel Islands are also poorly described.

Until recently, colonization events have been widely thought to occur from mainland to island, with islands thought of as "sinks" for colonizing species from mainland populations (Diamond, 1977; MacArthur & Wilson, 1963; Mayr, 1965). However, recent work has identified multiple island to mainland colonization events (Balke et al., 2009; Bellemain & Ricklefs, 2008; Filardi & Moyle, 2005; Funk & Burns, 2018; Jønsson, Bowie, et al., 2010; Jønsson et al., 2010). Although uncommon, several of these "upstream" colonization events from island to mainland have been followed by rapid population growth and are thought to have shaped some major biogeographic patterns such as the radiation of core Corvoidea (>700 species) from the proto‐Papuan archipelago to all other continents (Jønsson, Fabre, et al., 2010). In the current study, we investigate the Allen's Hummingbird (*Selasphorus sasin*), which includes a subspecies that recently colonized the southern California mainland and was a previous island endemic.

Allen's Hummingbird is found on both the Channel Islands and nearby mainland California. Allen's Hummingbird breeds in riparian habitats adjacent to sage scrub and forest edges along the mainland California coast (Jewett, 1929). There are two subspecies: one migratory (*S. s. sasin*) and one nonmigratory (*S. s. sedentarius*). Migratory Allen's Hummingbird has historically bred from Ventura County, in southern California (Grinnell & Miller, 1944), to extreme southern Oregon, while nonmigratory Allen's Hummingbird occurs on the Channel Islands and mainland southern California, from northern Baja California to Santa Barbara County along the coast, and inland to western Riverside County (Clark, 2017; Unitt, 2004; Wells & Baptista, 1979). Nonmigratory Allen's Hummingbird was previously endemic to the Channel Islands (Grinnell, 1939; Grinnell & Miller, 1944) and apparently colonized mainland southern California on the Palos Verdes Peninsula (located in Los Angeles County) in the 1960s (Allen et al., 2016; Bradley, 1980; Wells & Baptista, 1979). The most likely source of the colonizing population, based on geographic proximity, is Santa Catalina Island.

Recent work (Godwin et al., 2020) found no evidence of a founder effect on the mainland by nonmigratory Allen's Hummingbird, although this was based only on average nucleotide diversity (*π*) estimates. Further, Godwin et al. (2020) reported elevated levels of nucleotide diversity on the southern California mainland. However, their main focus was to evaluate the genetic relationships between nonmigratory and migratory Allen's Hummingbird, and sampling of nonmigratory Allen's Hummingbird was restricted to the coast on the mainland and the southern Channel Islands and did not include the northern Channel Islands, where bird species such as the Loggerhead Shrike (*Lanius ludovicianus*, Caballero & Ashley, 2011), Song Sparrow (*Melospiza melodia*, Wilson et al., 2015), and Spotted Towhee (*Pipilo maculatus*, Walsh, 2015) have exhibited high differentiation. While Godwin et al. (2020) analyzed the broader patterns of gene flow and divergence across Allen's Hummingbird (both subspecies) and Battey (2020) included both subspecies of Allen's as well as Rufous (*S. rufus*) Hummingbird, the present study explicitly focuses on the island–mainland colonization dynamics of nonmigratory Allen's Hummingbird.

Nonmigratory Allen's Hummingbird appears to be outcompeting migratory Allen's Hummingbird on mainland southern California and is rapidly expanding its range (Clark, 2017). A contributing factor to the success of nonmigratory Allen's Hummingbird may be a longer breeding season that results in higher fecundity (Clark, 2017; Clark & Mitchell, 2013). Exploitation of human‐modified habitat may also have a role in the rapid establishment and range expansion of nonmigratory Allen's Hummingbird (Clark, 2017). When nonmigratory Allen's Hummingbird colonized the Palos Verdes Peninsula in Los Angeles County, anthropogenic landscape alteration was already widespread in the area, and it was initially reported from within the urban landscape (Wells & Baptista, 1979). Nonmigratory Allen's Hummingbird is also more commonly observed within urban settings (i.e., in backyards) than migratory Allen's Hummingbird (Clark, 2017). Clark (2017) hypothesized that artificial features provided by human development such as hummingbird feeders and non‐native hummingbird‐friendly plants (i.e., Cape Honeysuckle, *Tecoma capensis*) support the success of nonmigratory Allen's Hummingbird. Further, abiotic factors might be associated with the establishment of nonmigratory Allen's Hummingbird on the mainland. For example, previous work found that rainfall and temperature variables correlated with the geographic distribution of Anna's (*Calypte anna*), Rufous, and Calliope (*S. calliope*) Hummingbird in western North America (Illán et al., 2014).

In the current study, we examine the colonization of the southern California mainland by nonmigratory Allen's Hummingbird to address four objectives. (a) We test for evidence of a founder effect via incorporation of genome‐wide estimates of *π* and Tajima's *D* (however, we acknowledge that any influence of a founder effect on these statistics may have been erased by ongoing gene flow with migratory Allen's Hummingbird and Channel Islands populations of nonmigratory Allen's Hummingbird). (b) We investigate how nucleotide diversity and genetic differentiation differ between nonmigratory Allen's Hummingbird on the mainland, the southern Channel Islands, and Santa Cruz Island, one of the northern Channel Islands. We also assess how patterns of differentiation and nucleotide diversity vary across the genome. (c) Nonmigratory Allen's Hummingbird has undergone a rapid, recent range expansion. Here, via ecological niche modeling, we investigate whether abiotic factors predict continued expansion by nonmigratory Allen's Hummingbird on the southern California mainland and compare projections of nonmigratory Allen's Hummingbird's niche to its migratory counterpart. Further, we test whether rainfall and temperature variables on the southern California mainland are associated with the establishment of nonmigratory Allen's Hummingbird from the Channel Islands. (d) We evaluate how genetic connectivity and differentiation of nonmigratory Allen's Hummingbird compares with other, similar studies of animal taxa breeding on the Channel Islands and adjacent mainland.

## 2 MATERIALS AND METHODS

### 2.1 Sampling

**FIGURE 1:**
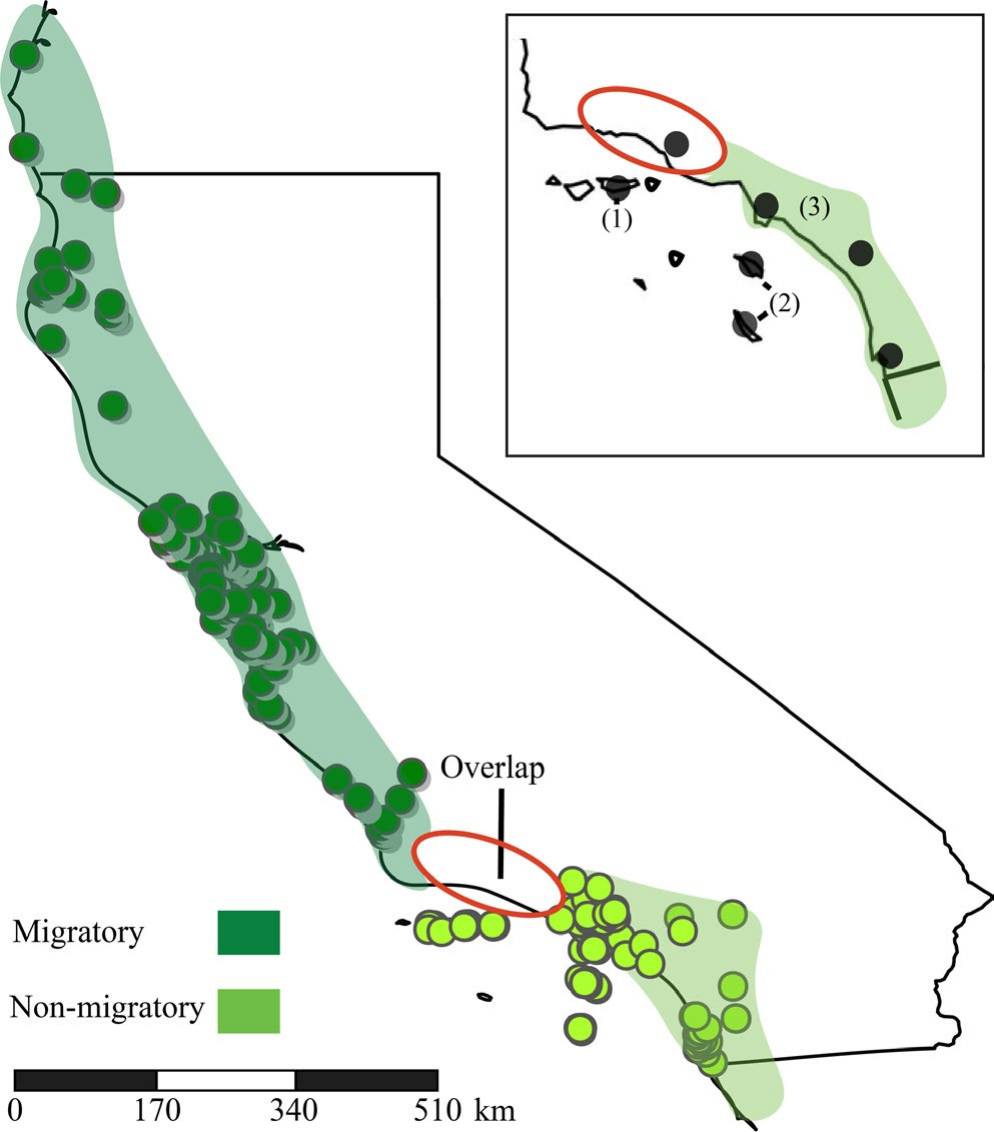
Approximate breeding ranges of migratory and nonmigratory Allen's Hummingbird, occurrence records used for ecological niche modeling, and sampling areas. Within the statewide map of California, each circle designates the GPS location of each occurrence record, while shaded areas denote the approximate breeding range of each subspecies. Within the inset map of southern California, numbers in parentheses denote genetic sampling locations of nonmigratory Allen's Hummingbird: (1) Santa Cruz Island, (2) Santa Catalina and San Clemente Island (southern Channel Islands), and (3) the mainland. Red circle indicates region where migratory and nonmigratory Allen's Hummingbird overlap in breeding range

We collected tissue (*N* = 17) and blood samples (*N* = 5; Figure 1; Appendix S1) from the Channel Islands (*N* = 10 individuals) and the southern California mainland (*N* = 12 individuals). All data in the current study were acquired from nonmigratory Allen's Hummingbird. Individuals included in the present study were gathered from the mainland in Los Angeles County (*N* = 5), as far south as San Diego County (*N* = 1), as far north as Santa Barbara County (*N* = 4), as far inland as Riverside County (*N* = 2), and from San Clemente (*N* = 1), Santa Catalina (*N* = 2), and Santa Cruz (*N* = 7) islands. Collection of all samples in the dataset occurred from March through May. All sampling we conducted was in compliance with the IACUC at the University of California, Riverside (protocols 20130018 and 20160039), USFWS permit #MB087454‐1, USGS Bird Banding Permit #23516, California Department of Fish and Wildlife permit #SC006598, and California State Parks permit #17‐820‐01 (Appendix S1).

### 2.2 DNA extraction and whole genome sequencing

Genomic DNA from tissues and dried blood spots were extracted using a Qiagen DNeasy Blood and Tissue Kit, following the recommendations of the manufacturer (Qiagen, Valencia, California, USA). Library preparation was based on a modified Nextera protocol (Baym et al., 2015). Whole genomes of all individuals were sequenced using an Illumina NextSeq 500 at the University of California, Riverside Genomics Core or an Illumina HiSeq X at Novogene, Inc. with an average coverage of 3.7x per sample (Appendix S2). Reads were aligned to the Anna's Hummingbird reference genome available on NCBI using the software package BWA v0.7 (Burrows‐Wheeler Aligner; Li & Durbin, 2009; NCBI Resource Coordinators, 2018). Variants were called using SAMtools v1.9 and BCFtools v1.9 (Li et al., 2009; Narasimhan et al., 2016). Using VCFtools v1.16 (Danecek et al., 2011), variants were filtered to have a minimum depth of one, to have been successfully genotyped in at least 50% of individuals, have a minimum mapping quality score of 30, and a minimum minor allele frequency of 0. We re‐analyzed samples with a minimum minor allele frequency of 0.05, and the patterns of results were unaffected (Appendix S3). After filtering, the dataset contained 1,770,572 SNPs.

### 2.3 Population structure

We investigated population structure present within the dataset using ADMIXTURE (Alexander et al., 2009). We evaluated clusters of K = 1–5, and the K value with the lowest cross‐validation error was chosen. To assess whether there was additional population structure, we implemented a principal component analysis (PCA), a model‐free method based on variation in allele frequencies, using PLINK v1.9 (Purcell et al., 2007). To ensure the data input into the PCA was independent (there were no spurious correlations among genomic variants), we pruned the dataset of linked variants by setting an r^2^ threshold of 0.1. Specifically, we pruned variables with an r^2^ greater than 0.1 within 50‐SNP windows to remove SNPs that were located close together on a given chromosome and in strong linkage disequilibrium (Purcell et al., 2007). We extracted PC coordinates for each individual and plotted the results using the "tidyverse" package v1.3.0 (Wickham et al., 2019) in R v3.5.2 (R Core Team, 2018) and R Studio v1.2.5 (R Studio Team, 2019).

### 2.4 Tajima's D

To investigate whether there was evidence for a founder effect in nonmigratory Allen's Hummingbird, we calculated the Tajima's *D* statistic in VCFtools v1.16, using a 5 kb sliding window for the mainland individuals in the dataset (Danecek et al., 2011; Tajima, 1989). A *D*‐value below zero would support a founder effect from the colonizing population of nonmigratory Allen's Hummingbird, followed by a population expansion. Using a sliding window approach, we compared values of Tajima's *D* across the genome.

### 2.5 Nucleotide diversity and differentiation

We calculated *π* in pixy v0.93, which incorporates variant and invariant sites to measure the degree of polymorphism within each population (Korunes & Samuk, 2020). Pixy overcomes an implicit simplifying assumption incorporated by most programs that use VCF files to calculate *π*, which typically only use variant sites and do not distinguish between invariant sites that are missing (but were genotyped) and sites that are truly missing from the dataset (Danecek et al., 2011). Such programs assume that missing sites are actually present in the dataset, but are invariant; thus, missing sites are added to the number of invariant sites, and π estimates are subsequently deflated (Korunes & Samuk, 2020). Nucleotide diversity was calculated for each chromosome using nonoverlapping sliding windows of 5 kb to compare genetic diversity estimates across the genome, and estimates were averaged within each window. Using a sliding window approach, we compared patterns of *π* across the genome. Evaluation of significance of π and *d_XY_* (see below) between Santa Cruz Island, the mainland, and San Clemente and Santa Catalina combined (hereafter called the "southern islands") was done by performing a Wilcoxon rank‐sum test in the R package tidyverse v1.3.0 (Wickham et al., 2019).

We calculated *d_XY_* in pixy to compare island and mainland population differentiation, which is an absolute measure of differentiation that calculates the average number of pairwise differences between sequences from two populations (Nei, 1987). Absolute differentiation measures are more reliable with full sequence data; thus, we incorporated variant and invariant sites into calculations of *d_XY_* (Cruickshank & Hahn, 2014). Using sliding windows of 5 kb, we compared patterns of *d_XY_* across the genome. For both π and *d_XY_* estimates, we constructed Manhattan plots in the R package qqman v0.1.4 (Turner, 2014) in R v3.5.2 (R Core Team, 2018) and R Studio v1.2.5 (R Studio Team, 2019).

Nucleotide diversity and differentiation statistics were analyzed such that each point on a Manhattan plot represents a log‐transformed p‐value of the *Z*‐score for the average values of π and *d_XY_* within a given 5 kb window. Before data were log‐transformed, a Bonferroni correction (calculated as 0.05/the total number of 5 kb windows) was applied to each p‐value. Outliers for π and *d_XY_* estimates are those which have a Bonferroni‐corrected p‐value (*p* < 2.45 × 10^–7^) that significantly deviates from the mean.

Migratory and nonmigratory Allen's Hummingbird form a zone of intergradation, with admixture detected from Santa Barbara to Los Angeles County (Godwin et al., 2020). To investigate the extent to which intergradation contributed to estimates of π, *d_XY_*, and Tajima's *D* on the mainland, we reran these analyses while including only nonmigratory Allen's Hummingbird individuals on the mainland as far as possible from the zone of intergradation reported by Godwin et al. (2020), from Riverside and San Diego County. Estimates of π, *d_XY_*, and Tajima's *D* calculations were unaffected (Appendix S4–S6). Thus, we incorporated results from all 22 individuals into all of our results. All results are reported as mean ± *SD*.

### 2.6 Ecological niche modeling

We implemented an ecological niche model using occurrence records of nonmigratory Allen's Hummingbird only on the Channel Islands to test whether rainfall and temperature variables are similar on the southern California mainland and Channel Islands. We evaluated whether the model showed high habitat suitability (defined as the capacity of the mainland to support the focal taxon) on the southern California mainland. We implemented 129 georeferenced occurrence records of field observations of Channel Island nonmigratory Allen's Hummingbird in VertNet (Constable et al., 2010). Only occurrence records from the breeding season (November–May for nonmigratory Allen's Hummingbird, March–June for migratory Allen's Hummingbird) were considered. We gathered current and historic climate data from the WorldClim version 2.0 database (Fick & Hijmans, 2017) in the form of 19 bioclimatic precipitation and temperature variables with a resolution of 2.5 arc minutes, translating to roughly five km (Phillips et al., 2006). To remove highly correlated variables, we followed Mason et al. (2014) to calculate pairwise correlation coefficients between variables and removed those which were redundant until no two variables had a correlation coefficient > 0.75. For a given pairwise comparison, the redundant variable that contributed the least to the model was removed via Jackknife analysis using the Cloglog output format in Maxent v3.4.1 (Phillips et al., 2006; Phillips & Dudik, 2008). As a result, we retained eight variables (Minitab 17 Statistical Software, 2017). Retained variables were annual mean temperature, mean diurnal range, isothermality, maximum temperature of the warmest month, mean temperature of the wettest quarter, mean temperature of the driest quarter, precipitation of the wettest month, and precipitation of the driest month. Using these variables, we modeled the niche of nonmigratory Allen's Hummingbird using Maxent version 3.4.1 (Phillips & Dudik, 2008). Models were based on 10 replicated runs with 25% of the data withheld for bootstrapping. We set the program to perform 5,000 iterations to allow model convergence with the data. We evaluated overall model performance using the area under the receiving operator characteristics curve (AUC), which ranges from 0.5 (randomness) to 1 (a perfect prediction).

We also investigated whether niche models project continued range expansion of nonmigratory Allen's Hummingbird on the southern California mainland and compared projections to migratory Allen's Hummingbird using occurrence records from their respective breeding ranges. To construct this niche model, we downloaded occurrence records based on georeferenced field observations by searching for both migratory and nonmigratory Allen's Hummingbird (as defined in Figure 1) in VertNet (Constable et al., 2010). Phenotypic differences between migratory and nonmigratory Allen's Hummingbird are only diagnosable in the hand on the basis of small mensural differences (Stiles, 1972), and there is likely error in using observational data. To ensure as much accuracy as possible, we classified occurrence records to the subspecies level on the basis of described distributions during the breeding season (Clark, 2017; Grinnell & Miller, 1944; Unitt, 2004; Wells & Baptista, 1979). Further, nonmigratory and migratory Allen's Hummingbird overlap in Ventura and Santa Barbara County during the breeding season, making delineation of subspecies identity difficult in these areas. To address this issue, we omitted observation records from Ventura and Santa Barbara County. The final dataset included 649 and 232 occurrence records across the breeding range of migratory and nonmigratory Allen's Hummingbird, respectively. In the niche model previously described, we restricted occurrence records for nonmigratory Allen's Hummingbird to the Channel Islands to see whether the model would predict colonization of the mainland. Here, we implemented occurrence records throughout the entire breeding range of both subspecies using the same data preparation and modeling methodology as described above.

## 3 RESULTS

### 3.1 Population structure

**FIGURE 2:**
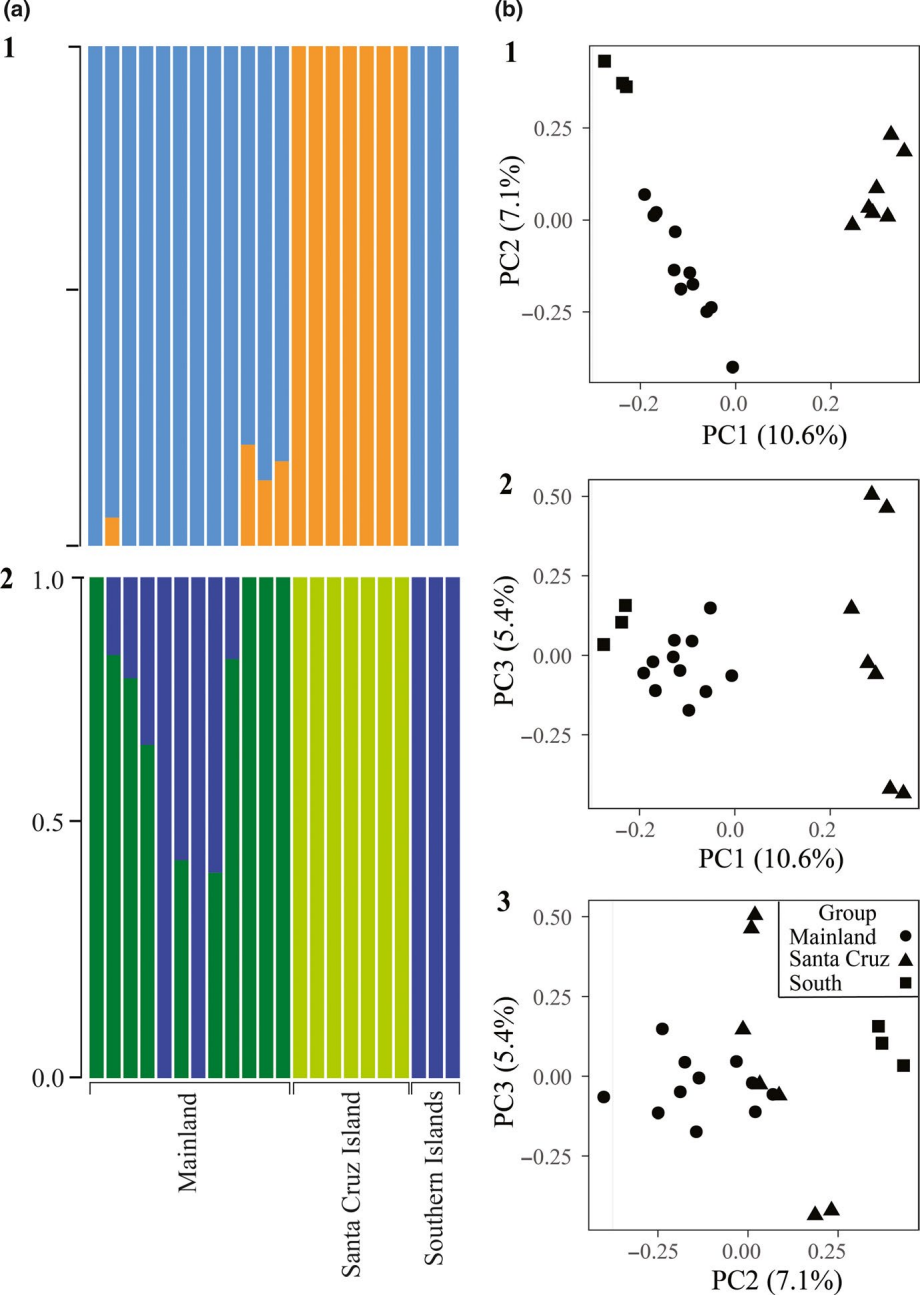
Population structure analysis for the southern Channel Islands, Santa Cruz Island, and the mainland. (a) ADMIXTURE plot for 1) K = 2 groups and 2) K = 3 groups. (b) Principal component analysis of the three groups across the first three principal components (PCs): 1) PC1 versus PC2 showed three main clusters, 2) PC1 versus PC3 showed five main clusters, 3) PC2 versus PC3 showed four main clusters

ADMIXTURE indicated that two clusters were best‐supported (K = 2, CV = 0.70), including one cluster of mainland and southern Channel Island individuals and one cluster comprised of Santa Cruz Island individuals (Figure 2a). K = 3 groups received the second‐highest support (CV = 0.86), with structure between the southern Channel Islands, Santa Cruz Island, and the mainland, although two mainland individuals clustered with the southern islands and six mainland individuals were admixed with the southern islands. PCA also supported K = 3 groups (see below, Figure 2a). Other clusters of K = 4 (CV = 1.13) and K = 5 (CV = 1.26) were not as well‐supported (Appendix S7).

PCA of whole genome data revealed population structure consistent with ADMIXTURE results that implemented K = 3 groups (Figure 2b). PC1 (10.6% of the variation) separated the southern Channel Islands, Santa Cruz Island, and the mainland across a continuum, with the most separation within the Santa Cruz Island cluster. PC2 (7.1% of the variation) fully distinguished the southern islands from the mainland and Santa Cruz Island. PC3 (5.4% of the variation) did not fully distinguish any group in any pairwise comparison but showed additional structure on Santa Cruz Island (Figure 2b).

### 3.2 Founder effects

**FIGURE 3:**
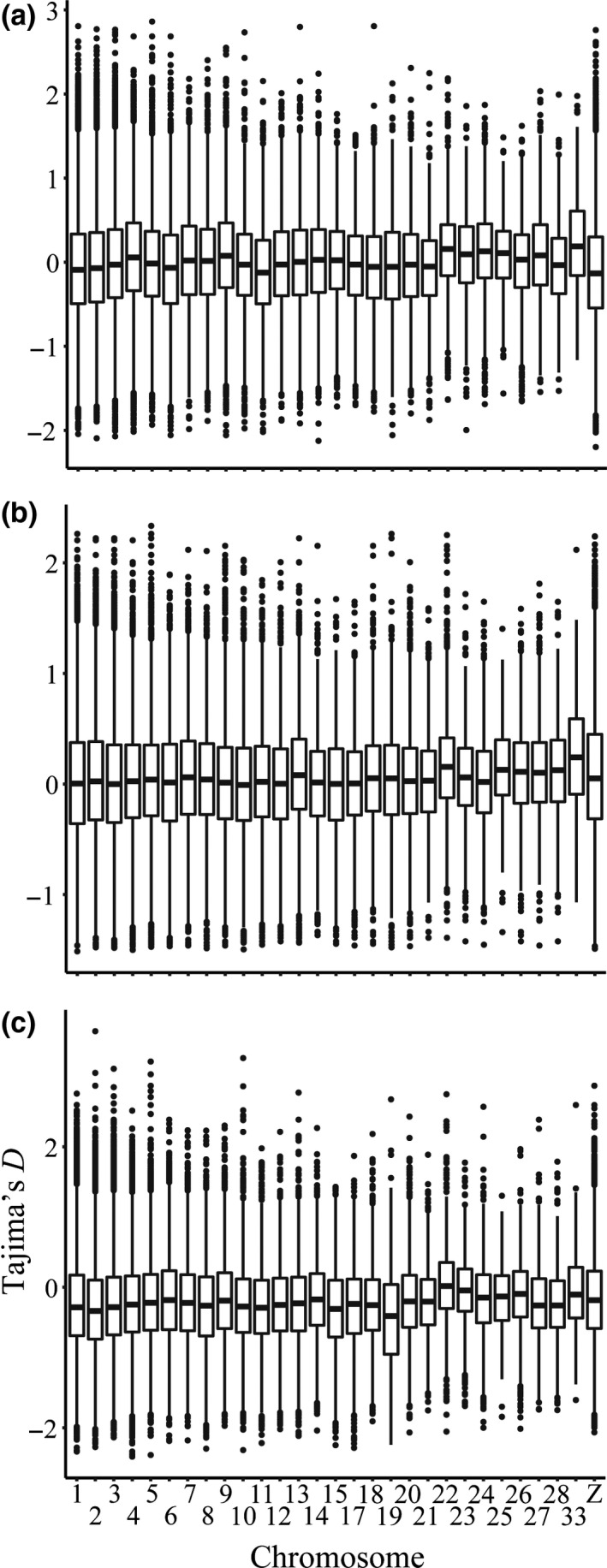
Tajima's *D*, by chromosome, for mainland nonmigratory Allen's Hummingbird for (a) Santa Cruz Island, (b) the southern Channel Islands, and (c) the mainland. Significantly positive and negative values of Tajima's *D* suggest departures from neutrality, while significantly negative values may also suggest a recent or ongoing population expansion

Tajima's *D* for mainland individuals did not differ from neutral expectations. Median values of Tajima's *D* were below zero on nearly every chromosome, although the mean of Tajima's *D* across the entire genome was not significantly different from zero (Tajima's *D*=−0.23 ± 0.62; Figure 3). Under the beta distribution, significant values of *D* (95% confidence) for mainland individuals would be below −1.77 or above 1.98 (Tajima, 1989). Based on this significance threshold, there was no evidence that a founder effect from the initial colonizing mainland population of Allen's Hummingbird preceded their rapid population expansion. Further, estimates of π were higher on the mainland than elsewhere (see below), thus, neither statistic supports a founder effect. For the southern Channel Islands and Santa Cruz Island, median Tajima's *D* values were also mostly below zero, with the mean not significantly different from zero in either case. There were several outlier loci present across the genome, most of which occurred within the mainland group, and were negative (725 negative outliers on the mainland, 162 on Santa Cruz Island, 0 on the southern islands; Table 1; Figure 3).

**TABLE 1:** Number of significant Tajima's *D* values on the autosomes and the Z chromosome for nonmigratory Allen's Hummingbird

Group	A (>1.98)	A (<−1.77)	Z (>1.98)	Z (<−1.77)
Santa Cruz Island	222	128	25	34
Southern Islands	58	0	8	0
Mainland	206	690	22	35

### 3.3 Nucleotide diversity and differentiation estimates

**FIGURE 4:**
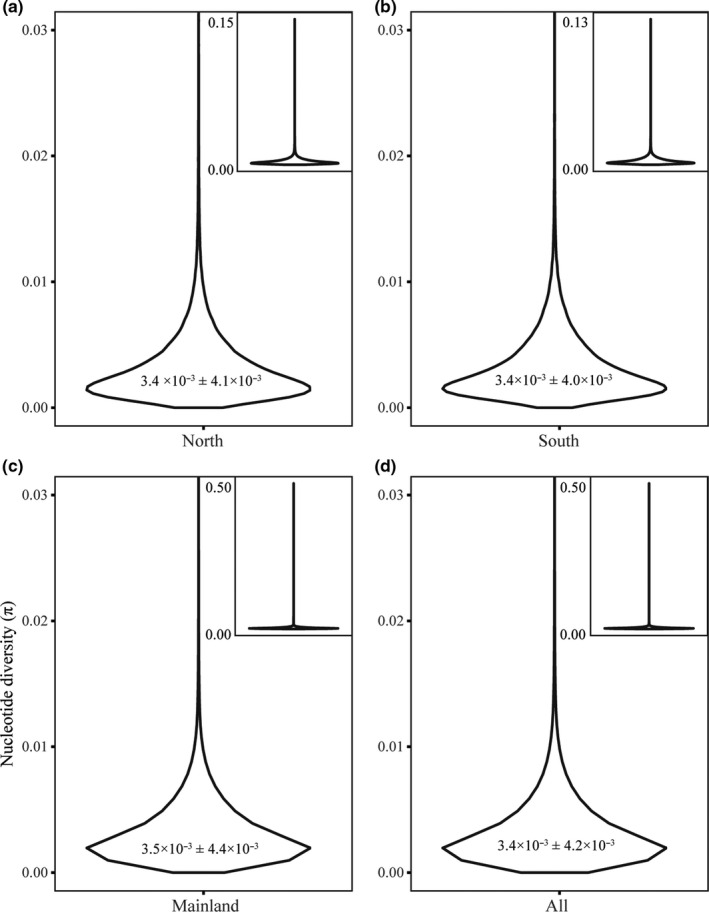
Genome‐wide nucleotide diversity (*π*) for nonmigratory Allen's Hummingbird, as follows: (a) Santa Cruz Island, (b) southern Channel Islands, (c) mainland, (d) all individuals in the dataset. Average *π* displayed within each violin plot. Outliers were cutoff in the main violin plot in each panel for clarity; the entire violin plot with outliers is included in the upper right corner of each panel

**FIGURE 5:**
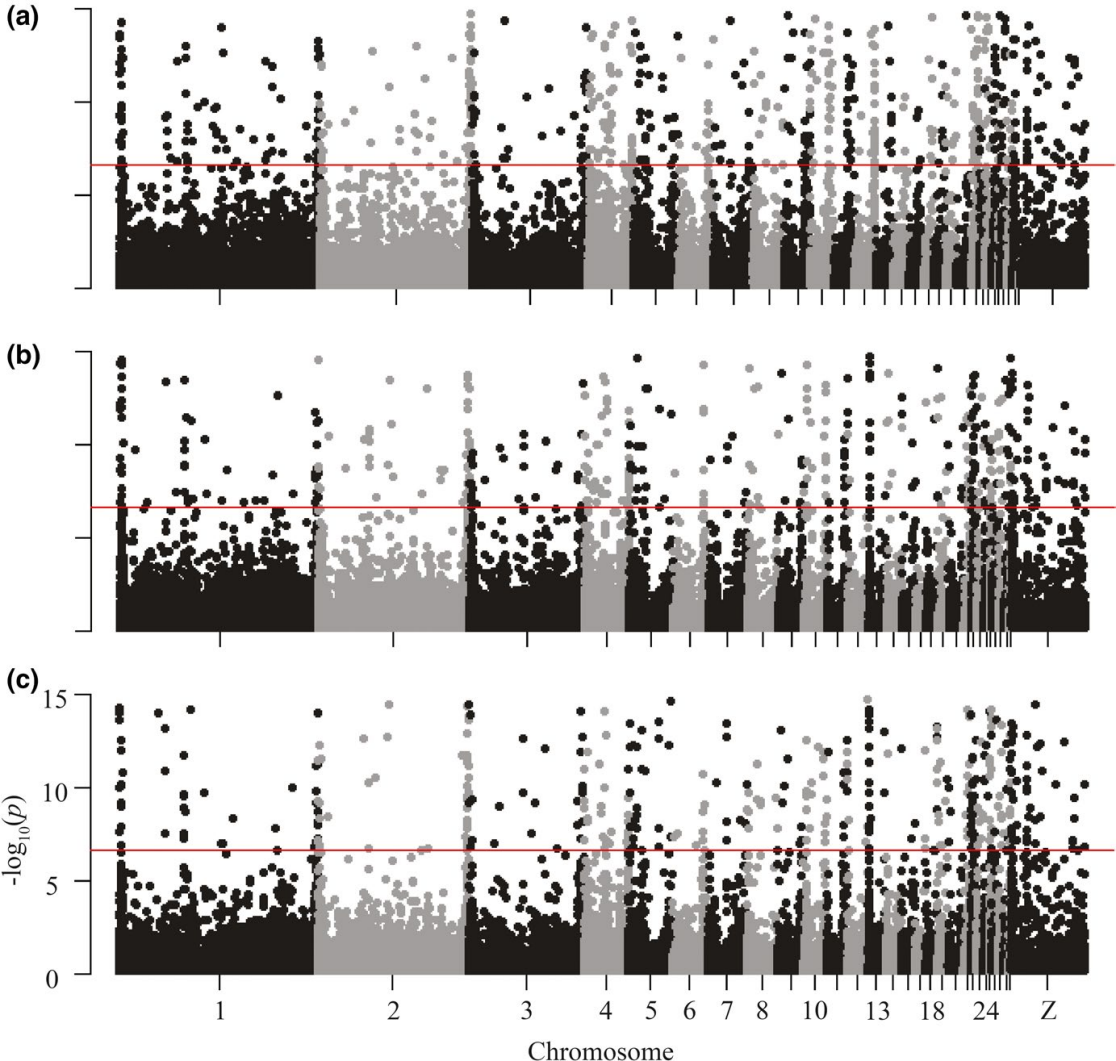
Log‐transformed p‐values of genome‐wide nucleotide diversity (*π*) for each nonoverlapping 5kb window, by chromosome, for nonmigratory Allen's Hummingbird for (a) Santa Cruz Island, (b) southern Channel Islands, and (c) the mainland. The red genome‐wide significance line indicates the Bonferroni‐corrected significance threshold of 2.45 × 10^–7^

Measures of genetic differentiation (π and *d_XY_*) mostly overlapped across the southern Channel Islands, Santa Cruz Island, and the mainland. Autosomal nucleotide diversity was comparable across all groups (Santa Cruz Island, 3.4 × 10^–3^ ± 3.5 × 10^–3^; the southern islands, 3.4 × 10^–3^ ± 3.2 × 10^–3^; the mainland, 3.4 × 10^–3^ ± 3.3 × 10^–3^; Table 2; Figure 4). Despite high overlap, average π was significantly higher on the mainland than on the southern islands and Santa Cruz Island (Wilcoxon rank‐sum test, *p* < .05), while average π did not differ significantly between the southern islands and Santa Cruz Island (Wilcoxon rank‐sum test, *p* > .05). Estimates of π on the Z chromosome (the sex chromosome) were nearly half of estimates on autosomes (Table 2). There were many outliers distributed across the 5 kb windows of the genome on the Channel Islands and the mainland, and Santa Cruz Island had the most (966 outliers; Table 2; Figure 5). Most outliers were concentrated toward the outer edges of each chromosome (Figure 5).

**TABLE 2:** Differentiation (*d_XY_*) and nucleotide diversity (π) on the autosomes and the Z chromosome for nonmigratory Allen's Hummingbird

	Autosomes	Z chromosome
Pairwise comparison (*d_XY_*)		
Santa Cruz Island versus mainland	3.6 × 10^–3^ ± 3.3 × 10^–3^	1.8 × 10^–3^ ± 2.4 × 10^–3^
Santa Cruz Island versus south	3.6 × 10^–3^ ± 3.3 × 10^–3^	1.7 × 10^–3^ ± 2.3 × 10^–3^
Southern islands versus mainland	3.4 × 10^–3^ ± 2.9 × 10^–3^	1.6 × 10^–3^ ± 2.1 × 10^–3^
Nucleotide diversity (π)		
Entire dataset (*N* = 22)	3.4 × 10^–3^ ± 3.4 × 10^–3^	1.8 × 10^–3^ ± 2.5 × 10^–3^
Santa Cruz Island (*N* = 7)	3.4 × 10^–3^ ± 3.5 × 10^–3^	1.8 × 10^–3^ ± 2.7 × 10^–3^
Southern Channel Islands (*N* = 3)	3.4 × 10^–3^ ± 3.2 × 10^–3^	1.8 × 10^–3^ ± 2.4 × 10^–3^
Mainland (*N* = 12)	3.4 × 10^–3^ ± 3.3 × 10^–3^	1.7 × 10^–3^ ± 2.4 × 10^–3^

Differentiation was low across all pairwise group comparisons. Because *d_XY_* is a between‐population estimate of π, similarly to estimates of π, values of *d_XY_* were nearly halved on the Z chromosome in comparison to autosomes (Table 2). Values of *d_XY_* ranged from 3.2 × 10^–3^ ± 3.6 × 10^–3^ to 3.5 × 10^–3^ ± 3.9 × 10^–3^. Average *d_XY_* for comparisons involving Santa Cruz Island (Santa Cruz Island versus the mainland and Santa Cruz Island versus the southern islands) were significantly higher than *d_XY_* between the southern islands and the mainland (Wilcoxon rank‐sum test, *p* < .05). There was no significant difference between the means of Santa Cruz Island versus the mainland and Santa Cruz Island versus the southern islands (Wilcoxon rank‐sum test, *p* > .05). Thus, intergroup comparisons (*d_XY_*) were similar to intragroup comparisons (π), with two exceptions: higher *d_XY_* with both pairwise comparisons involving Santa Cruz Island and elevated π on the mainland. Outside of these two exceptions, individuals between islands and the mainland differed little from individuals within the islands and mainland. However, there were many outlier loci that were more differentiated than others (relatively higher *d_XY_*) across the genome, and most outliers were concentrated toward the outer edges of each chromosome (Table 3; Figure 7).

**TABLE 3:** The number of significantly differentiated (*d_XY_*) loci and the number of significantly elevated estimates of nucleotide diversity (*π*) on the autosomes and the Z chromosome for nonmigratory Allen's Hummingbird. Significance levels were based on a Bonferroni‐corrected significance threshold of 2.45 × 10^–7^

	Autosomes	Z
Pairwise comparison (*d_XY_*)		
Santa Cruz Island versus mainland	753	91
Santa Cruz Island versus southern islands	709	86
Southern islands versus mainland	712	91
Nucleotide diversity (*π*)		
Santa Cruz Island	904	62
Southern Islands	665	83
Mainland	654	80

### 3.4 Ecological niche models

The ecological niche model for nonmigratory Allen's Hummingbird that tested whether abiotic factors are similar on the Channel Islands and the mainland identified similar climatic data on the islands and the mainland California coast. Values reflecting suitable habitat spanned from moderate (0.50) to moderately high (0.75) from San Diego to Los Angeles County, moderate in Santa Barbara County, and moderate to extremely high (0.95) in San Luis Obispo County (Figure 8c). The average AUC score across all variables was 0.99, indicating high support for the model. Thus, climate data are similar between the mainland and Channel Islands.

The ecological niche model of nonmigratory Allen's Hummingbird that evaluated the potential for range expansion identified additional suitable habitat beyond its current breeding range. The average AUC scores for migratory and nonmigratory Allen's Hummingbird were 0.98 and 0.97, respectively, indicating high support for each model. Visual inspection of the niche models suggested that nonmigratory Allen's Hummingbird had slightly lower habitat suitability in San Luis Obispo County than migratory Allen's Hummingbird, and migratory Allen's Hummingbird had only slightly suitable habitat at the northern limit of its current distribution in extreme northern California and southern Oregon (Figure 8). Models also suggested the presence of slightly suitable habitat for migratory Allen's Hummingbird south of Los Angeles County, beyond its southern range limit (Figure 8a). Further, there was suitable habitat beyond the current described breeding range of nonmigratory Allen's Hummingbird, with highly suitable habitat available north into San Luis Obispo County, extending to the San Francisco Bay Area (Figure 8b). Additionally, the models predicted a continued inland expansion (beyond the areas it occupies currently) of nonmigratory Allen's Hummingbird, showing suitable habitat east of San Diego toward the San Diego and Imperial County border near the Anza Borrego Desert, and north of the Los Angeles basin toward Antelope Valley, Hesperia, and Victorville in the Los Angeles and San Bernardino County high desert, and into Kern County near Bakersfield (Figure 8b).

## 4 DISCUSSION

### 4.1 Founder effects

Colonizing populations are prone to founder effects. Nonmigratory Allen's Hummingbird, a subspecies previously endemic to the Channel Islands, apparently colonized mainland southern California from the southern Channel Islands some time before 1970 (Allen et al., 2016; Bradley, 1980; Wells & Baptista, 1979). Given the recent colonization of nonmigratory Allen's Hummingbird on the mainland, a founder effect in the mainland population was expected. However, we did not detect a founder effect, as evidenced by a nonsignificant deviation of Tajima's *D* from zero and elevated π on the mainland, similar to Godwin et al. (2020). In the event of a bottleneck, we would expect lower π within the founding population. If there were initial founder effects on the mainland, two ongoing phenomena likely eroded them: gene flow from migratory Allen's Hummingbird and continued gene flow with the Channel Islands since the initial colonization event.

Loci with significantly negative Tajima's *D* scores may be indicative of a population expansion (Tajima, 1989). On the mainland, we identified 725 negative outliers (compared to 0 on the southern islands and 162 on Santa Cruz Island) that may reflect the documented population expansion of mainland nonmigratory Allen's Hummingbird via the presence of many low frequency polymorphisms (Table 1; Figure 3). However, further analysis of individual loci interpreted via Tajima's *D* is needed, as demographic phenomena are known to skew the values of Tajima's *D* for single loci (Kelley et al., 2006). Further, the southern islands had the fewest *Tajima's D* outliers: There were 0 outliers for negative values of Tajima's *D* and 66 positive outlier loci. Estimates of Tajima's *D* can be biased with lower sample sizes (Marroni et al., 2011). Thus, low sample size on the southern islands (*N* = 3) could explain the relatively small number of total Tajima's *D* outliers found on the southern islands (Table 1).

### 4.2 Nucleotide diversity and differentiation

Levels of π for nonmigratory Allen's Hummingbird are comparable to other bird species (Figure 4), which commonly have π values from 10^–2^ to 10^–3^, but vary, spanning from passerines (with a range from 1.8 × 10^–3^ to 1.1 × 10^–2^) to landfowl (with a range from 1.1 × 10^–3^ to 5.0 × 10^–3^; Ellegren, 2013). Estimates of π were highest on the mainland. Nucleotide diversity is a reflection of effective population size (*N_e_*; Ellegren, 2009), which might suggest a higher *N_e_* of mainland nonmigratory Allen's Hummingbird relative to the islands. The elevated π observed on the mainland could be due to ongoing gene flow with migratory Allen's Hummingbird and nonmigratory Allen's Hummingbird from the southern Channel Islands, as gene flow can increase diversity estimates (Godwin et al., 2020).

Generally, autosomes are expected to be more polymorphic than sex chromosomes in natural populations, where the ratio of π of sex chromosomes to autosomes is expected to be about 0.75 (Balakrishnan & Edwards, 2009; Corl & Ellegren, 2012). Deviations from this pattern are likely when the number of reproducing males and females differs (i.e., in polygamous mating systems, when a single male mates with several females, the number of sex chromosomes relative to autosomes in the breeding population decreases relative to numbers acquired during random mating; Ellegren, 2009). Thus, in polygamous species, the sex chromosome to autosome ratio is often less than 0.75 (Corl & Ellegren, 2012). Hummingbirds are generally thought to be polygynous (Schuchmann, 1999), that is, some males may not reproduce, yielding a lower *N_e_* of males relative to females. Consistent with this expectation, we found that the Z chromosome to autosome ratio in the current dataset was 0.53 (Table 2).

Differentiation estimates mirrored nucleotide diversity estimates: *d_XY_* was also about twice as large on the autosomes as on the Z chromosome. The low estimates of *d_XY_* on the Z chromosome relative to the autosomes (and low *d_XY_* overall) may reflect that nonmigratory Allen's Hummingbird populations have diverged little overall; in this scenario, *π* and *d_XY_* are expected to be similar (Dutoit et al., 2017). In undiverged populations, *π* and *d_XY_* are expected to largely reflect ancestral polymorphism. As reproductive isolation or time since divergence increases, *d_XY_* is expected to increase, while *π* is generally expected to remain constant (Henderson & Brelsford, 2020). In addition to the finding that Santa Cruz Island is the most diverged of the groups of populations included in our study (see below), we also observed increased *d_XY_* on the Z chromosome when comparing Santa Cruz Island to the mainland and Santa Cruz Island to the southern islands (Table 2). A small ancestral population size or low *N_e_* might also lead to low *d_XY_* on the Z chromosome relative to the autosomes (Presgraves & Yi, 2009). Further, we observed elevated *π* and *d_XY_* on chromosomal edges (Figure 5; Figure 7). Some bird species have higher recombination rates at the edges of chromosomes, which are expected to lead to higher diversity estimates (unpbl. data; Backstrӧm et al., 2010). When divergence is low, elevated *d_XY_* along chromosome edges may also be observed given the expectations of similarity between *π* and *d_XY_* in this scenario (Henderson & Brelsford, 2020).

Demography, which includes divergence events and the timing of such events (as explained above), migration, and changes in population size over time, can have large effects on genomic data (Pyhäjärvi et al., 2007). Estimates of *π* and *d_XY_* varied widely throughout the genome across all populations, and there were many outlier loci present on every chromosome (Figures 5 and 7). While values of π and *d_XY_* are known to fluctuate (Puzey et al., 2017), demographic processes increase the variance observed in estimates such as *π* and *d_XY_* (Depaulis et al., 2003). However, delineating specific demographic events (and the timing of such events) requires rigorous testing of alternative demographic scenarios. The data did not support a founder effect of nonmigratory Allen's Hummingbird on the mainland; however, a founder effect on the mainland represents only one of many possible events across evolutionary time.

**FIGURE 6:**
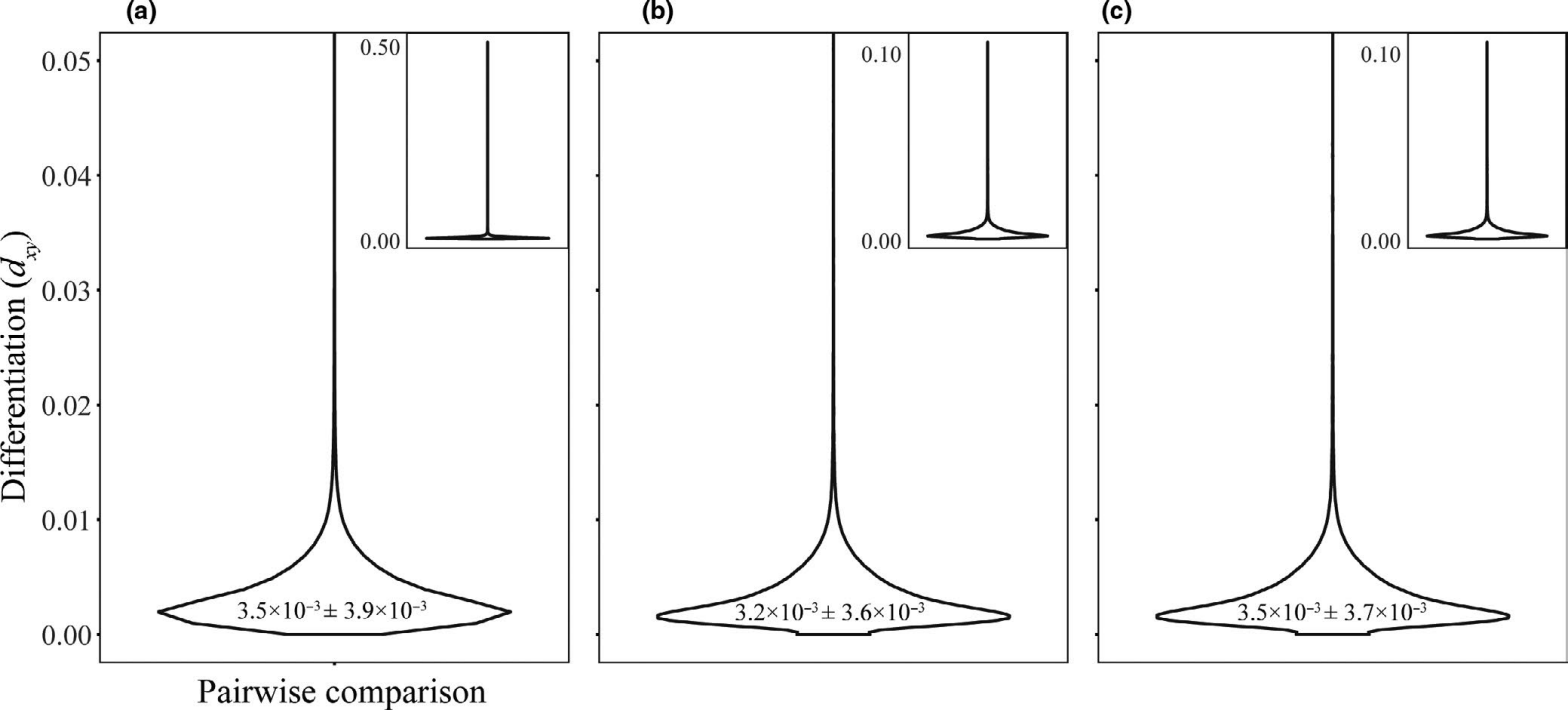
Pairwise genome‐wide differentiation (*d_XY_*) for nonmigratory Allen's Hummingbird, as follows: (a) Santa Cruz Island versus the mainland, (b) southern Channel Islands versus the mainland, (c) Santa Cruz Island versus the southern Channel Islands. Average *d_XY_* displayed within each violin plot. Outliers were cutoff in the main violin plot in each panel for clarity; the entire violin plot with outliers is included in the upper right corner of each panel

**FIGURE 7:**
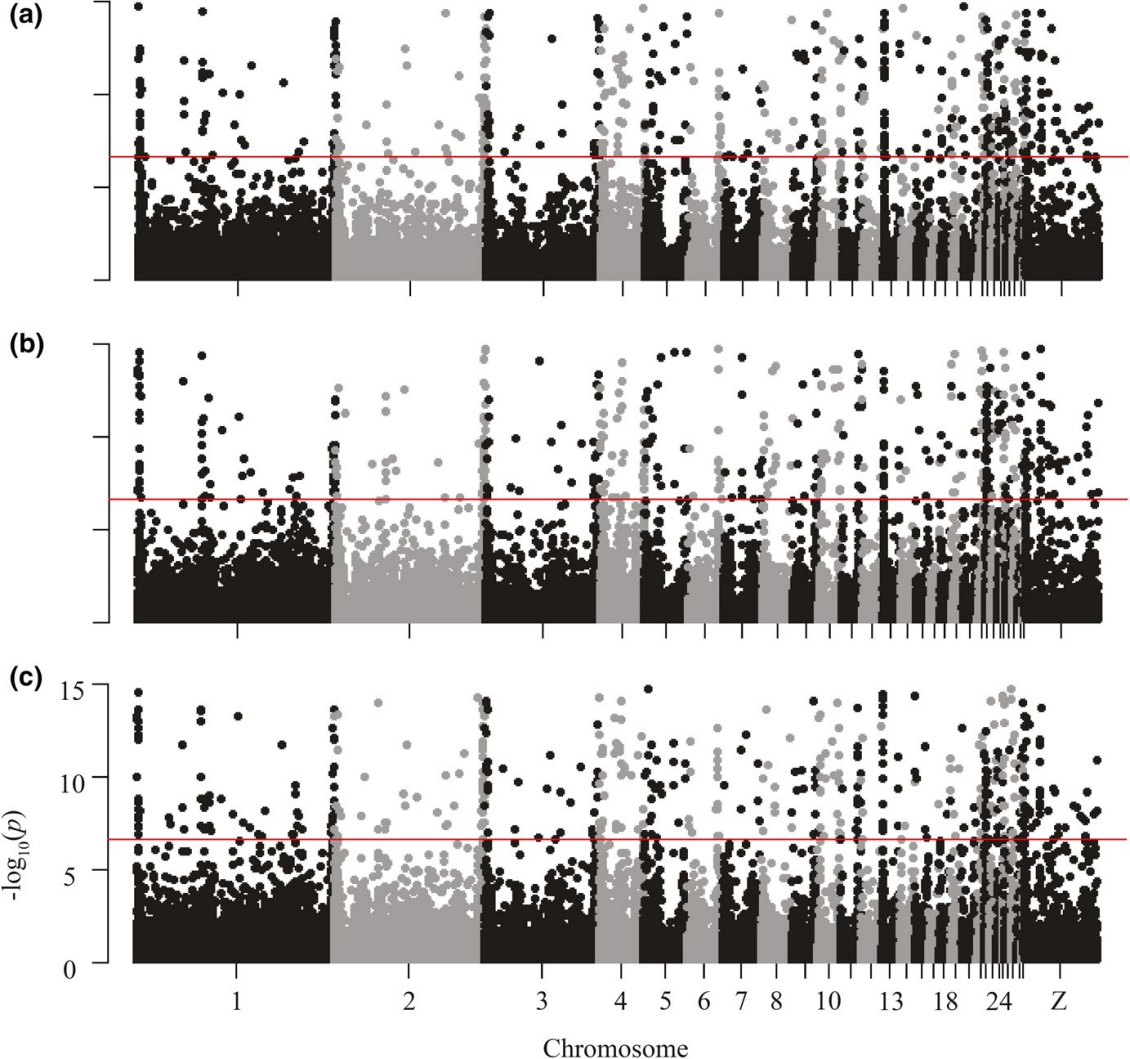
Log‐transformed p‐values of genome‐wide differentiation (*d_XY_*) for each nonoverlapping 5kb window, by chromosome, for all nonmigratory Allen's Hummingbird. Pairwise comparisons of groups displayed as follows: (a) Santa Cruz Island versus the mainland, (b) the southern Channel Islands versus Santa Cruz Island, (c) the mainland versus the southern Channel Islands. The red genome‐wide significance line indicates the Bonferroni‐corrected significance threshold of 2.45 × 10^–7^

Although divergence estimates were low, it is possible that some loci with elevated *d_XY_* (Figure 7) indicate the presence of locally beneficial variants (Yu et al., 2003). Average *d_XY_* of pairwise comparisons that included Santa Cruz Island (southern islands versus Santa Cruz Island, the mainland versus Santa Cruz Island) were higher than average *d_XY_* of the southern islands versus the mainland. The loci with the highest differentiation occurred when comparing Santa Cruz Island to the mainland, and the lowest pairwise comparison was between the mainland and southern Channel Islands (Figure 6). While the elevated estimates of π on the mainland likely reflect ongoing gene flow from migratory Allen's Hummingbird and the southern Channel Islands, increased *d_XY_* (in addition to the population structure identified via ADMIXTURE and PCA) on Santa Cruz Island appears to reflect its relative isolation from the other groups. Values of *d_XY_* are in agreement with prior *F_ST_* estimates, which, within nonmigratory Allen's Hummingbird, were highest for all pairwise comparisons involving Santa Cruz Island (Santa Cruz Island versus the mainland and Santa Cruz Island versus the southern islands; unpbl. data). Further, the structure identified on Santa Cruz Island is similar to other Channel Island taxa that show that differentiated populations on the Channel Islands are typically found on the northern Channel Islands (see below; Figure 7).

Estimates of *d_XY_* and π indicated that individuals differed little within a given group (π) and between groups (*d_XY_*). However, the best‐supported ADMIXTURE analysis identified two clusters: Santa Cruz Island and the southern islands combined with the mainland. The second‐best ADMIXTURE analysis, which identified three clusters, was consistent with PCA results: PC1 fully separated Santa Cruz Island, the southern islands, and the mainland, and PC2 separated the southern islands from the other two groups (Figure 2).

Godwin et al. (2020) identified two distinct groups consistent with the two subspecies of Allen's Hummingbird in addition to one intermediate group that resulted from admixture of these subspecies. However, Godwin et al. (2020) did not include samples from the northern Channel Islands, which may be an important source of differentiation: work across a suite of organisms has found elevated differentiation and low gene flow between the northern islands and southern islands and the northern islands and mainland (see below). Thus, the additional structure found by PCA in the present study was unidentifiable using the sampling design of Godwin et al. (2020; Figure 3).

### 4.3 Ecological niche modeling

**FIGURE 8:**
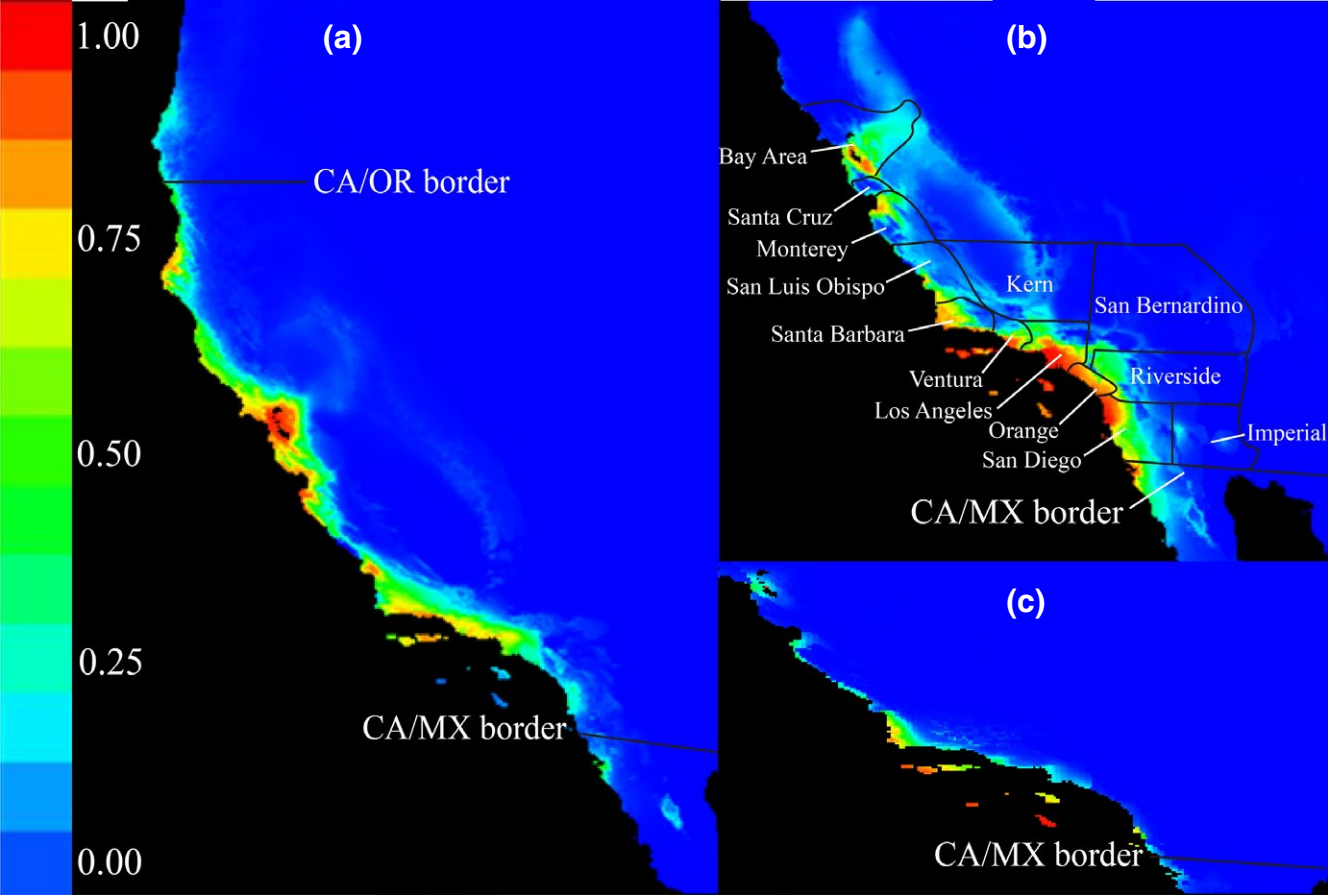
Ecological niche models, which evaluated (a) the current suitable habitat for migratory Allen's Hummingbird, (b) nonmigratory Allen's Hummingbird, and (c) whether rainfall and temperature variables are similar on the southern California mainland and Channel Islands, showing projected suitable habitat on the southern California mainland, and supporting its establishment on the mainland. All models are based on rainfall and temperature data from the WorldClim database (Fick & Hijmans, 2017). Warmer colors indicate more highly suitable habitat. State and country borders are indicated on each map; other text (b) indicates notable California counties to clarify where nonmigratory Allen's Hummingbird is predicted to further expand its range: north of Santa Barbara, into San Luis Obispo County (and possibly beyond, as far north as the San Francisco Bay Area), near the San Diego/Imperial County border, into San Bernardino County toward Hesperia and Victorville in the high desert, and near the Los Angeles/Kern County border, into Lancaster and toward Bakersfield

Ecological niche models suggested that the southern California mainland has similar rainfall and temperature as the Channel Islands, especially in coastal areas near the islands (Figure 8c). In ecological niche models, island species such as nonmigratory Allen's Hummingbird often exemplify "Wallace's Dream," meaning that, prior to colonization of the mainland, the distribution of nonmigratory Allen's Hummingbird was limited by dispersal and not a lack of favorable habitat (Escobar et al., 2018; Saupe et al., 2012). Under this condition, the actual ecological niche of the species may be greater in extent than the model shows (Escobar et al., 2018; Saupe et al., 2012). With this caveat in mind, the niche models presented here imply that nonmigratory Allen's Hummingbird may continue to expand north to San Luis Obispo County (and possibly beyond, to the San Francisco Bay Area) and inland near the Anza Borrego Desert in San Diego County, toward the Antelope Valley and Hesperia in the Los Angeles high desert and adjacent parts of San Bernardino County, and into Kern County, toward Bakersfield (Figure 8b). If the niche models were overly conservative due to a dispersal limitation on nonmigratory Allen's Hummingbird, this subspecies might have an unoccupied niche that extends beyond the regions identified by our models.

According to our niche models, migratory and nonmigratory Allen's Hummingbird share suitable habitat in southern California (Figure 8). The overlap in suitable habitat we report is supported by the admixture found between nonmigratory and migratory Allen's Hummingbird on the southern California mainland (Godwin et al., 2020). Nonmigratory Allen's Hummingbird has rapidly expanded its range ever since it colonized the southern California mainland (Bradley, 1980; Clark, 2017), and niche models showed suitable habitat for nonmigratory Allen's Hummingbird as far north as the San Francisco Bay area (Figure 8b). Although niche models for migratory Allen's Hummingbird generally showed higher habitat suitability north of Santa Barbara County, nonmigratory Allen's Hummingbird has a longer breeding season and may have an advantage within urbanized areas and might be encroaching into the range of migratory Allen's Hummingbird (Clark, 2017; Clark & Mitchell, 2013). Recently, nonmigratory Allen's Hummingbird has spread into urban areas that were formerly arid sage scrub in southern California (Riverside County), a habitat in which neither subspecies of Allen's Hummingbird is historically known (Bradley, 1980; Clark, 2017; Grinnell & Miller, 1944). Clark (2017) hypothesized that there is a subtle ecological or behavioral difference between migratory and nonmigratory Allen's Hummingbird that allows nonmigratory Allen's Hummingbird to better‐exploit urban habitat, although this requires formal evaluation. Further, a longer breeding season may result in elevated levels of fecundity, which increases the potential for range expansion (Jan et al., 2019). With a breeding season lasting from November through May, one nonmigratory Allen's Hummingbird female has enough time to fledge at least four nests per year, compared to a maximum potential of two nests per year by a single migratory Allen's Hummingbird female that breeds from March through June (Clark, 2017; Clark & Mitchell, 2013).

### 4.4 Phylogeographic patterns across the channel Islands

A growing body of literature has investigated the population genetics of vertebrate species on the Channel Islands relative to the mainland. Here, we discuss how these histories compare to our findings with regard to Allen's Hummingbird. The islands themselves have never been attached to the mainland, and during the mid‐Pleistocene, most of the Channel Islands were submerged until sea levels began to lower, although higher elevation areas of Santa Catalina, Santa Cruz, and Santa Rosa Island may have remained above water (Johnson, 1977). The northern islands were united into one large island by the late Pleistocene (125,000–750,000 years ago) as a result of lowered sea levels, which narrowed the distance from the mainland to about six km and presented a possible colonization window for several taxa (Johnson, 1977).

The relationships of populations on the Channel Islands relative to the mainland vary among species. Some species on the mainland are weakly differentiated and show high levels of gene flow between the mainland and Channel Islands (Horned Lark, Mason et al., 2014; Orange‐crowned Warbler, *Oreothlypis celata*, Sofaer et al., 2012; Hanna et al., 2019; nonmigratory Allen's Hummingbird). In contrast, others species have island populations that are strongly differentiated from their mainland counterparts, indicating little to no gene flow between the mainland and the Channel Islands (Island Scrub‐Jay, *Aphelcoma insularis*, Delaney & Wayne, 2005; Loggerhead Shrike, Caballero & Ashley, 2011; Spotted Towhee, Walsh, 2015; Song Sparrow, Wilson et al., 2015). Further, populations on the northern Channel Islands tend to show high differentiation relative to other populations (Loggerhead Shrike, Caballero & Ashley, 2011; Song Sparrow, Wilson et al., 2015; Spotted Towhee, Walsh, 2015), as we show here for nonmigratory Allen's Hummingbird (Figure 6).

Nonavian vertebrates generally show stronger differentiation and fewer colonization events between the Channel Islands and the mainland (Deer Mouse, *Peromyscus maniculatus*, Ashley & Willis, 1987; Wayne et al., 1991; Island Night Lizard, *Xantusia riversiana*, Rice, 2017). Nonavian vertebrates, similar to birds, are often the most differentiated on the northern Channel Islands (Deer Mouse, Ashley & Willis, 1987; California Channel Island Fox, *Urocyon littoralis*, Gilbert et al., 1990; Pergams et al., 2000). Two exceptions to high northern island differentiation, likely due to recent colonization, are found in the weakly differentiated Channel Island Spotted Skunk (*Spilogale gracilis amphiala*, Floyd et al., 2011) and the Western Harvest Mouse (*Reithrodontomys megalotis*, Ashley, 1989).

Northern Channel Island populations are usually more closely related to each other than any other population. The northern islands were connected during the LGM; thus, the close relationships of populations on the northern islands correspond to geologic history (Schoenherr et al., 1999). However, at least in the case of nonmigratory Allen's Hummingbird, colonization of the mainland from the southern islands could mask much of the signal of connectivity between the northern islands and the mainland. The initial colonization event from the southern Channel Islands, followed by the rapid range expansion of the colonizing population, would likely lead to comparatively high levels of gene flow between the southern islands and mainland relative to estimates between the northern islands and the mainland.

Genetic drift is one potential explanation for the relative isolation of nonmigratory Allen's Hummingbird on Santa Cruz Island. For example, drift is documented to have contributed to elevated genetic divergence of the Loggerhead Shrike and Song Sparrow on the northern islands (Caballero & Ashley, 2011; Wilson et al., 2015). Recent management effort on Santa Cruz Island to enhance the habitat for avifauna was followed by a colonization of the island by the Song Sparrow, and possibly the subsequent drift effects observed. Drift might also be observed if species were present in small numbers before management action took place and benefitted from these efforts (Van Vuren, 2013).

Several factors may explain the contrasting patterns seen among the different taxa studied on the Channel Islands. The varying levels of differentiation seen across species between the islands and the mainland could be due to gene flow, dispersal ability, recent colonization, multiple colonization events, timing of colonization, incomplete lineage sorting, and even the choice of genetic markers and sampling design. Thus, some observed differences may be due to evolutionary phenomena and others due to discordance in data analysis and marker use (markers span from microsatellites, to nuclear genes, to mitochondrial genes, to whole genomes). Due to the idiosyncratic nature of island colonization, more taxa need to be studied using a comprehensive approach before broad conclusions can be drawn regarding Channel Island–mainland patterns and relationships.

## 5 DATA ACCESSIBILITY STATEMENT

All original genomic data used in this study are publicly available on Dryad at https://doi.org/10.5061/dryad.cnp5hqc30.

## CONFLICT OF INTEREST

The authors claim no competing interests.

## AUTHOR CONTRIBUTION

**Brian Myers:** Conceptualization (lead); Data curation (lead); Formal analysis (lead); Investigation (lead); Methodology (lead); Project administration (lead); Resources (lead); Software (lead); Validation (lead); Visualization (lead); Writing‐original draft (lead); Writing‐review & editing (lead). **Kevin Burns:** Conceptualization (supporting); Funding acquisition (lead); Investigation (supporting); Methodology (supporting); Resources (supporting); Supervision (supporting); Visualization (supporting); Writing‐review & editing (supporting). **Christopher Clark:** Conceptualization (supporting); Formal analysis (supporting); Funding acquisition (lead); Methodology (supporting); Resources (lead); Supervision (lead); Writing‐review & editing (supporting). **Alan Brelsford:** Conceptualization (supporting); Data curation (supporting); Formal analysis (supporting); Funding acquisition (lead); Methodology (supporting); Resources (lead); Software (supporting); Supervision (supporting); Writing‐review & editing (supporting).

## Supporting information

Appendix S1‐S7

Appendix S4

Appendix S5

Appendix S6

Appendix S7
