# Supplementary material for: The population genetics of nonmigratory Allen’s Hummingbird (Selasphorus sasin sedentarius) following a recent mainland colonization
Source: Ecol Evol. 2021 Jan 20;11(4):1850–65. doi: 10.1002/ece3.7174 (PMC7882939; doi:10.1002/ece3.7174)
Supplement: Supplementary file 1 — Appendix S1‐S7 [file ECE3-11-1850-s001.docx]

Appendix 1. Coordinates, locality, and county for all samples (N=22). Vouchered tissue samples are indicated with an associated museum: San Diego State University Museum of Biodiversity (SDSU), San Diego Natural History Museum (SDNHM), and Museum of Vertebrate Zoology (MVZ). Individuals with a band number were released after blood sample collection; all other samples are from collected individuals. Data collection dates are in month/day/year format.

| Band or museum ID | Collection  date | Locality | County, State | Latitude (N) | Longitude (W) |
| --- | --- | --- | --- | --- | --- |
| SDSU2980 | 3/8/2017 | Point Mugu State Park | Los Angeles, CA | 34°4'21.00" | 119°0'49.80” |
| E05849 | 3/17/2017 | University of California, Santa Barbara | Santa Barbara, CA | 34°24'32.61" | 119°52'41.28” |
| SDNHM51665 | 4/5/2007 | San Clemente Island | Los Angeles, CA | 32°55'01.14” | 118°30'38.34” |
| SDSU3098 | 2/2/2016 | Santa Catalina Island | Los Angeles, CA | 33°21'23.17" | 118°26'22.81” |
| SDSU3097 | 2/2/2016 | Santa Catalina Island | Los Angeles, CA | 33°21'23.17” | 118°26'22.81” |
| E05980 | 3/13/2017 | Point Mugu State Park | Los Angeles, CA | 34°4'21.00” | 119°0'49.80” |
| MVZ183549 | 3/27/2006 | Santa Cruz Island Reserve | Santa Barbara, CA | 33°59'45.80” | 119°43'30.46” |
| MVZ183551 | 3/27/2006 | Santa Cruz Island Reserve | Santa Barbara, CA | 33°59'45.80” | 119°43'30.46” |
| MVZ183552 | 3/27/2006 | Santa Cruz Island Reserve | Santa Barbara, CA | 33°59'45.80” | 119°43'30.46” |
| MVZ183553 | 3/27/2006 | Santa Cruz Island Reserve | Santa Barbara, CA | 33°59'45.80” | 119°43'30.46” |
| MVZ183554 | 3/28/2006 | Santa Cruz Island Reserve | Santa Barbara, CA | 33°59'45.80” | 119°43'30.46” |
| MVZ183555 | 3/29/2006 | Santa Cruz Island Reserve | Santa Barbara, CA | 33°59'45.80” | 119°43'30.46” |
| MVZ183556 | 3/29/2006 | Santa Cruz Island Reserve | Santa Barbara, CA | 33°59'45.80” | 119°43'30.46” |
| SDNHM50767 | 11/25/2002 | Tarzana | Los Angeles, CA | 34°10'28.65” | 118°33'11.62” |
| SDNHM51657 | 3/13/2007 | Hancock Park | Los Angeles, CA | 34°04'04.04” | 118°19'57.27” |
| SDNHM53030 | 5/4/2011 | Encinitas | San Diego, CA | 33°02'20.04” | 117°17'17.88” |
| SDSU3101 | 1/19/2016 | University of California, Riverside | Riverside, CA | 33°58'23.44" | 117°19'21.80" |
| SDSU3099 | 12/21/2015 | University of California, Riverside | Riverside, CA | 33°58'23.44" | 117°19'21.80" |
| E05850 | 3/16/2017 | University of California, Santa Barbara | Santa Barbara, CA | 34°24'32.61” | 119°52'41.28” |
| SDSU2981 | 3/9/2017 | Point Mugu State Park | Los Angeles, CA | 34°4'21.00” | 119°0'49.80” |
| E05999 | 3/17/2017 | University of California, Santa Barbara | Santa Barbara, CA | 34°24'32.61” | 119°52'41.28” |
| E05848 | 3/16/2017 | University of California, Santa Barbara | Santa Barbara, CA | 34°24'32.61” | 119°52'41.28” |

Appendix 2. Library prep information for all samples (N=22). Vouchered tissue samples are indicated with an associated museum: San Diego State University Museum of Biodiversity (SDSU), San Diego Natural History Museum (SDNHM), and Museum of Vertebrate Zoology (MVZ). Individuals with a band number were released after blood sample collection; all other samples are from collected individuals.

| Band or museum ID | County, State | Sex | Sequencer | Read length | Depth |
| --- | --- | --- | --- | --- | --- |
| SDSU2980 | Los Angeles, CA | M | HiSeq X, Novogene | 150bp PE | 4.17 |
| E05849 | Santa Barbara, CA | M | HiSeq X, Novogene | 150bp PE | 2.55 |
| SDNHM51665 | Los Angeles, CA | M | HiSeq X, Novogene | 150bp PE | 2.27 |
| SDSU3098 | Los Angeles, CA | M | HiSeq X, Novogene | 150bp PE | 6.01 |
| SDSU3097 | Los Angeles, CA | M | HiSeq X, Novogene | 150bp PE | 2.73 |
| E05980 | Los Angeles, CA | M | HiSeq X, Novogene | 150bp PE | 5.40 |
| MVZ183549 | Santa Barbara, CA | M | HiSeq 4000, UC Berkeley | 150bp PE | 2.00 |
| MVZ183551 | Santa Barbara, CA | M | HiSeq 4000, UC Berkeley | 150bp PE | 3.36 |
| MVZ183552 | Santa Barbara, CA | M | HiSeq 4000, UC Berkeley | 150bp PE | 2.73 |
| MVZ183553 | Santa Barbara, CA | M | HiSeq 4000, UC Berkeley | 150bp PE | 2.88 |
| MVZ183554 | Santa Barbara, CA | M | HiSeq 4000, UC Berkeley | 150bp PE | 3.09 |
| MVZ183555 | Santa Barbara, CA | M | HiSeq 4000, UC Berkeley | 150bp PE | 2.42 |
| MVZ183556 | Santa Barbara, CA | M | HiSeq 4000, UC Berkeley | 150bp PE | 3.54 |
| SDNHM50767 | Los Angeles, CA | M | HiSeq X, Novogene | 150bp PE | 2.88 |
| SDNHM51657 | Los Angeles, CA | F | HiSeq X, Novogene | 150bp PE | 2.94 |
| SDNHM53030 | San Diego, CA | F | HiSeq X, Novogene | 150bp PE | 2.60 |
| SDSU3101 | Riverside, CA | M | HiSeq X, Novogene | 150bp PE | 6.22 |
| SDSU3099 | Riverside, CA | M | HiSeq X, Novogene | 150bp PE | 4.71 |
| E05850 | Santa Barbara, CA | M | HiSeq X, Novogene | 150bp PE | 6.22 |
| SDSU2981 | Los Angeles, CA | M | HiSeq X, Novogene | 150bp PE | 4.17 |
| E05999 | Santa Barbara, CA | M | HiSeq X, Novogene | 150bp PE | 6.64 |
| E05848 | Santa Barbara, CA | F | HiSeq X, Novogene | 150bp PE | 2.80 |

Appendix 3. Average Tajima's *D*, differentiation (*d_XY_*) and nucleotide diversity (π) for non-migratory Allen's Hummingbird with a minor allele frequency of 0.05.

| Statistic | Mean ± S.D. |
| --- | --- |
| Tajima's *D* | 0.37 ± 0.43 |
| d_XY_ |  |
| Southern islands versus mainland | 3.2 × 10^-3^ ± 3.6 × 10^-3^ |
| Southern islands versus Santa Cruz Island | 3.5 × 10^-3^ ± 3.7 × 10^-3^ |
| Mainland versus Santa Cruz Island | 3.5 × 10^-3^ ± 3.9 × 10^-3^ |
| π |  |
| Santa Cruz Island | 3.4 × 10^-3^ ± 4.1 × 10^-3^ |
| Southern islands | 3.4 × 10^-3^ ± 4.0 × 10^-3^ |
| Mainland | 3.5 × 10^-3^ ± 4.4 × 10^-3^ |
| All individuals | 3.4 × 10^-3^ ± 4.2 × 10^-3^ |

**
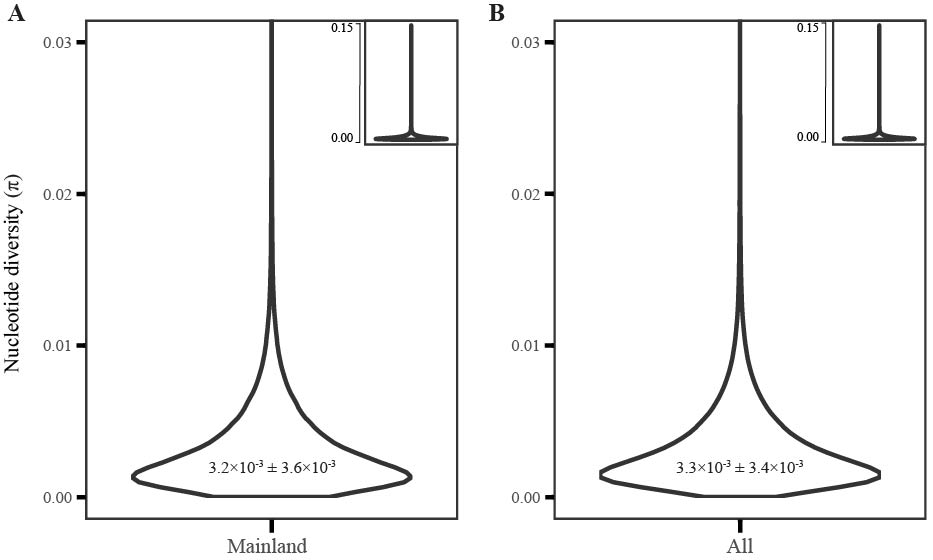
**

Appendix 4. Genome-wide nucleotide diversity (π) for non-migratory Allen's Hummingbird, including (A) mainland individuals, and (B) all individuals in the dataset, excluding individuals from Santa Barbara and Los Angeles County. Average π displayed within each violin plot. Outliers were cut off in the main violin plot in each panel for clarity; the entire violin plot with outliers is included in the upper right corner of each panel.


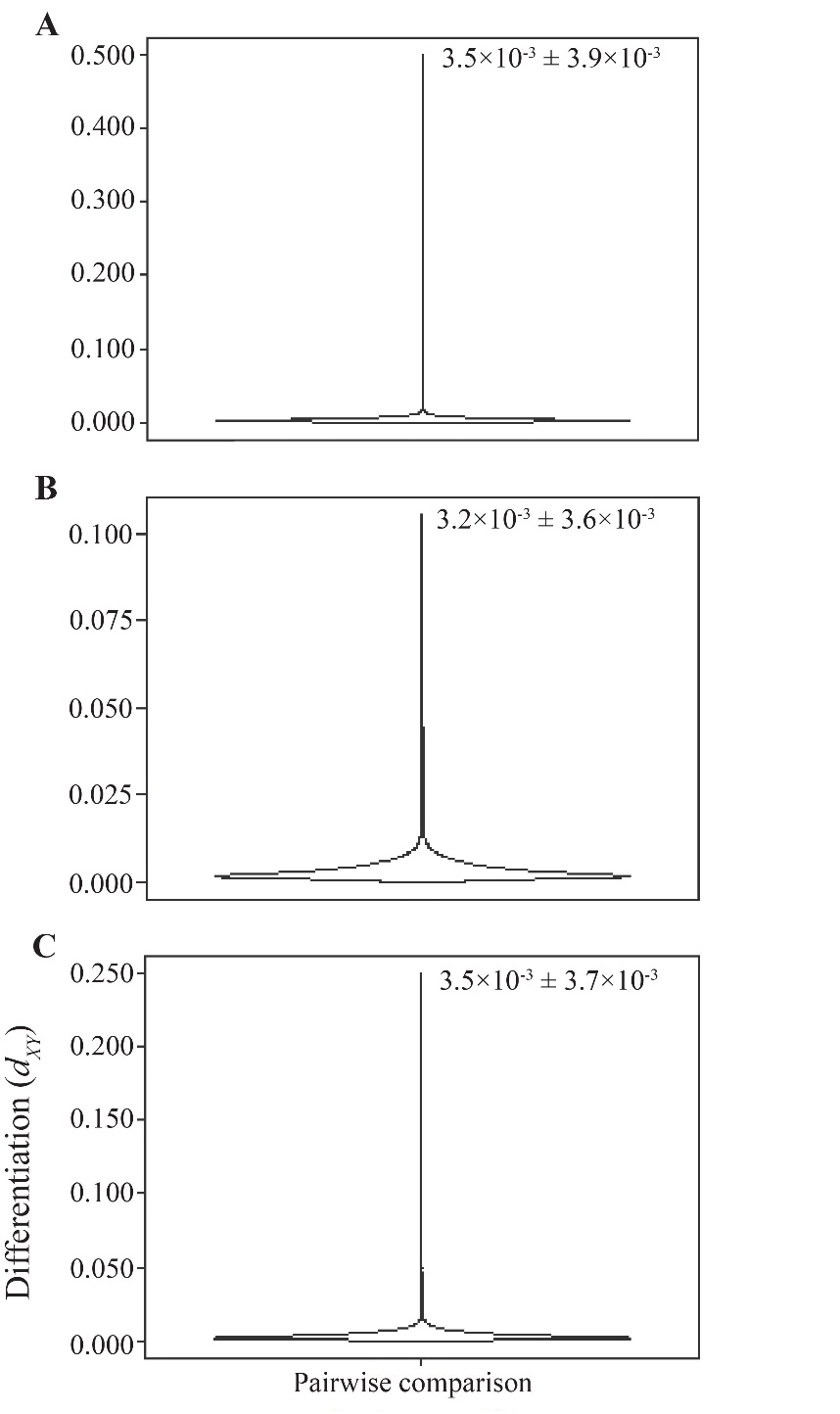


Appendix 5. Pairwise genome-wide differentiation (*d_XY_*) for non-migratory Allen's Hummingbird, excluding individuals from Santa Barbara and Los Angeles County, as follows: (A) Santa Cruz Island versus the mainland, (B) southern Channel Islands versus the mainland, (C) Santa Cruz Island versus the southern Channel Islands. Average *d_XY_* displayed within each violin plot. Outliers were cut off in the main violin plot in each panel for clarity; the entire violin plot with outliers is included in the upper right corner of each panel.


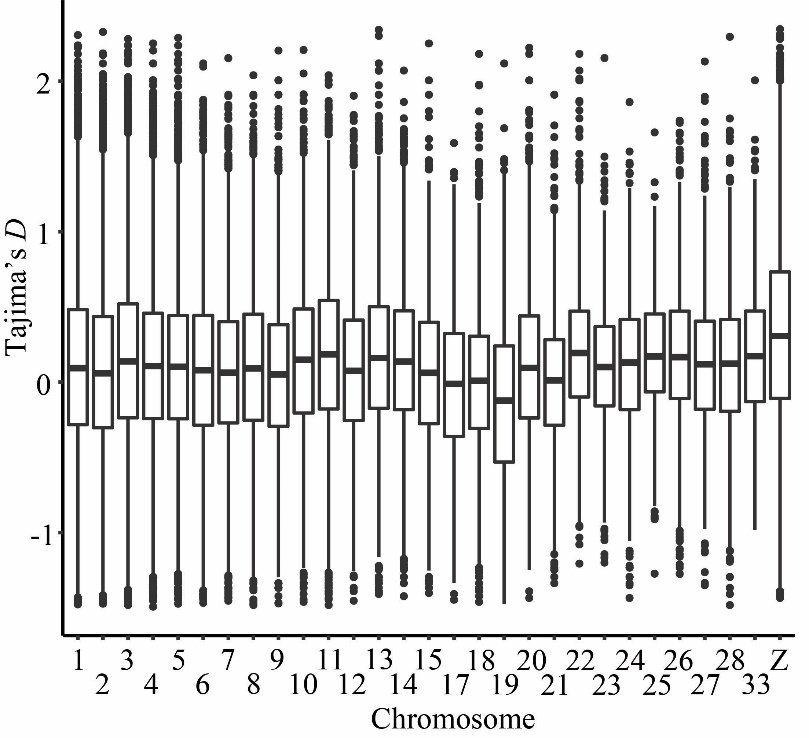


Appendix 6. Tajima's *D*, by chromosome, for mainland non-migratory Allen's Hummingbird, excluding individuals from Santa Barbara and Los Angeles County. Significantly positive and negative values of Tajima's *D* suggest departures from neutrality, while significantly negative values may also suggest a recent or ongoing population expansion.


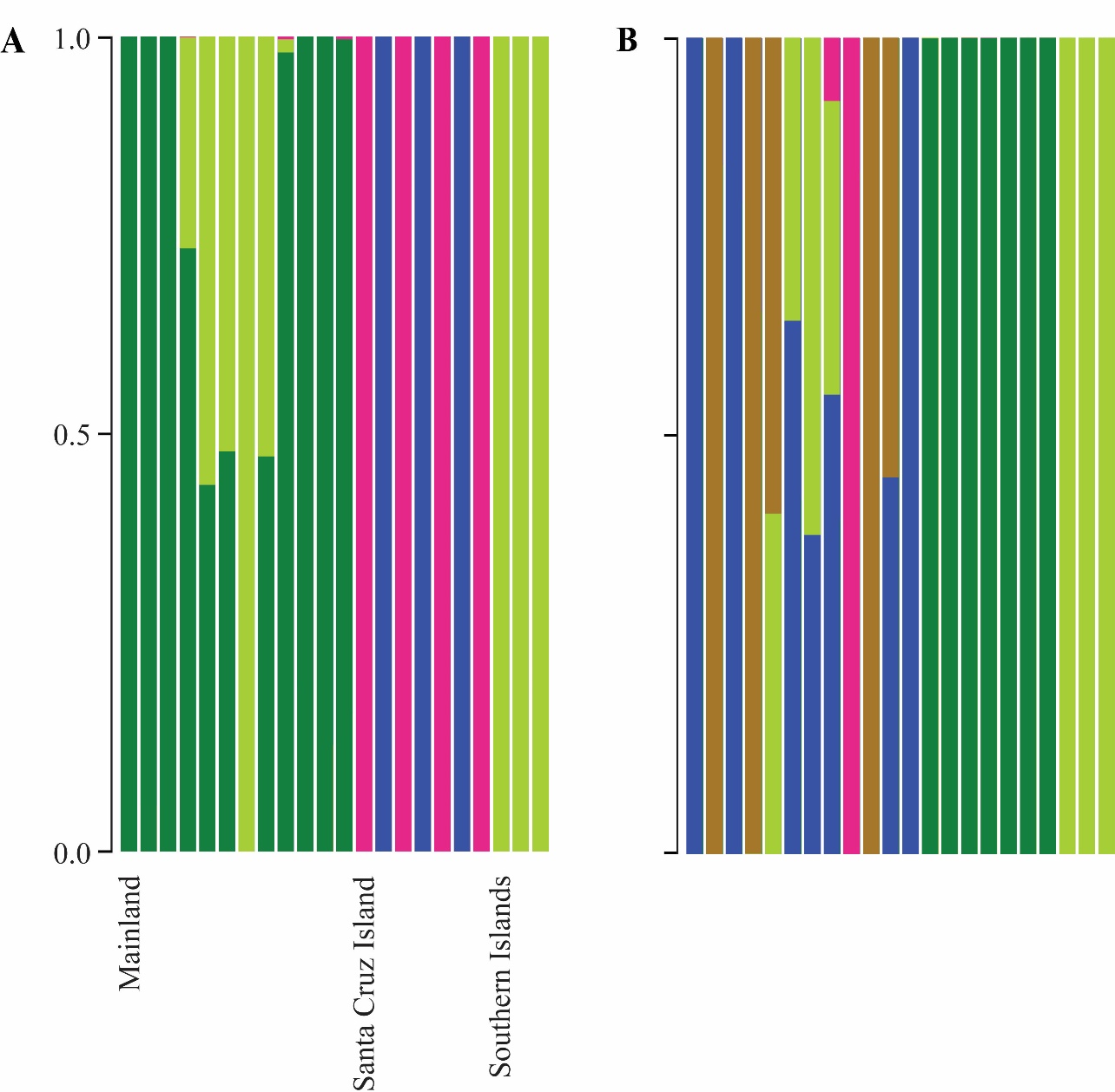


Appendix 7. ADMIXTURE analysis for the southern Channel Islands, Santa Cruz Island, and the mainland for A) K=4 groups, where the mainland and southern islands formed their own clusters, with admixture present on the mainland with the southern islands, two clusters present within Santa Cruz Island, and B) K=5 groups, where there are three clusters on the mainland with high admixture between clusters and with the southern islands, and separate clusters on Santa Cruz Island and the southern islands.
