# Supplementary figures and images for: The population genetics of nonmigratory Allen’s Hummingbird (Selasphorus sasin sedentarius) following a recent mainland colonization
Source: Ecol Evol. 2021 Jan 20;11(4):1850–65. doi: 10.1002/ece3.7174 (PMC7882939; doi:10.1002/ece3.7174)

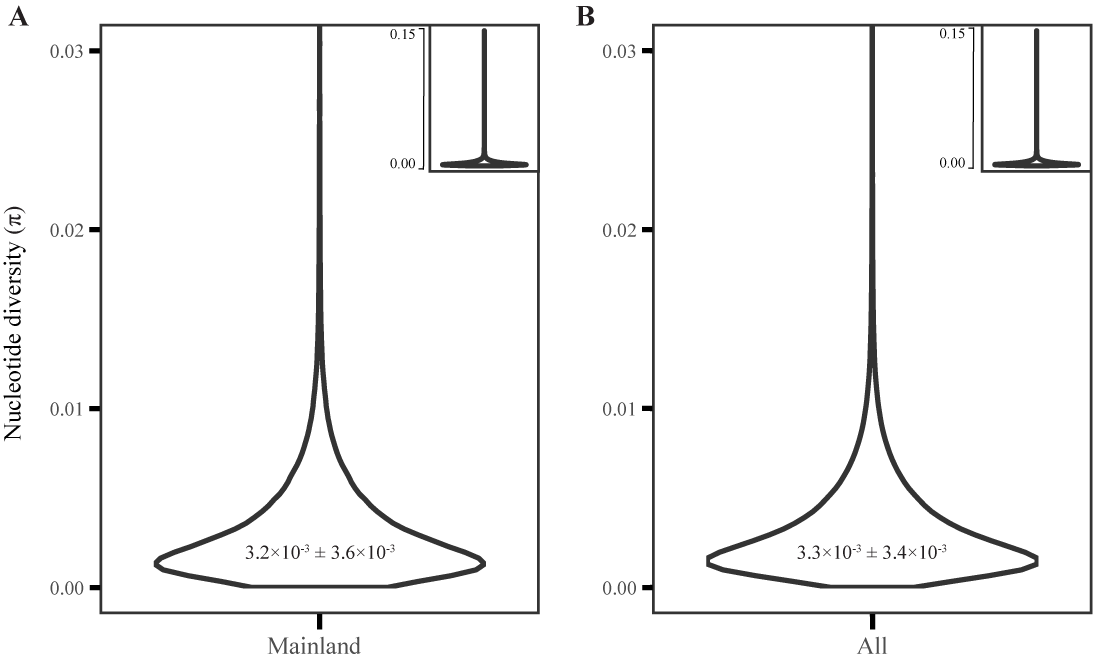

Supplement: Supplementary file 2 — Appendix S4 [file ECE3-11-1850-s002.tif]

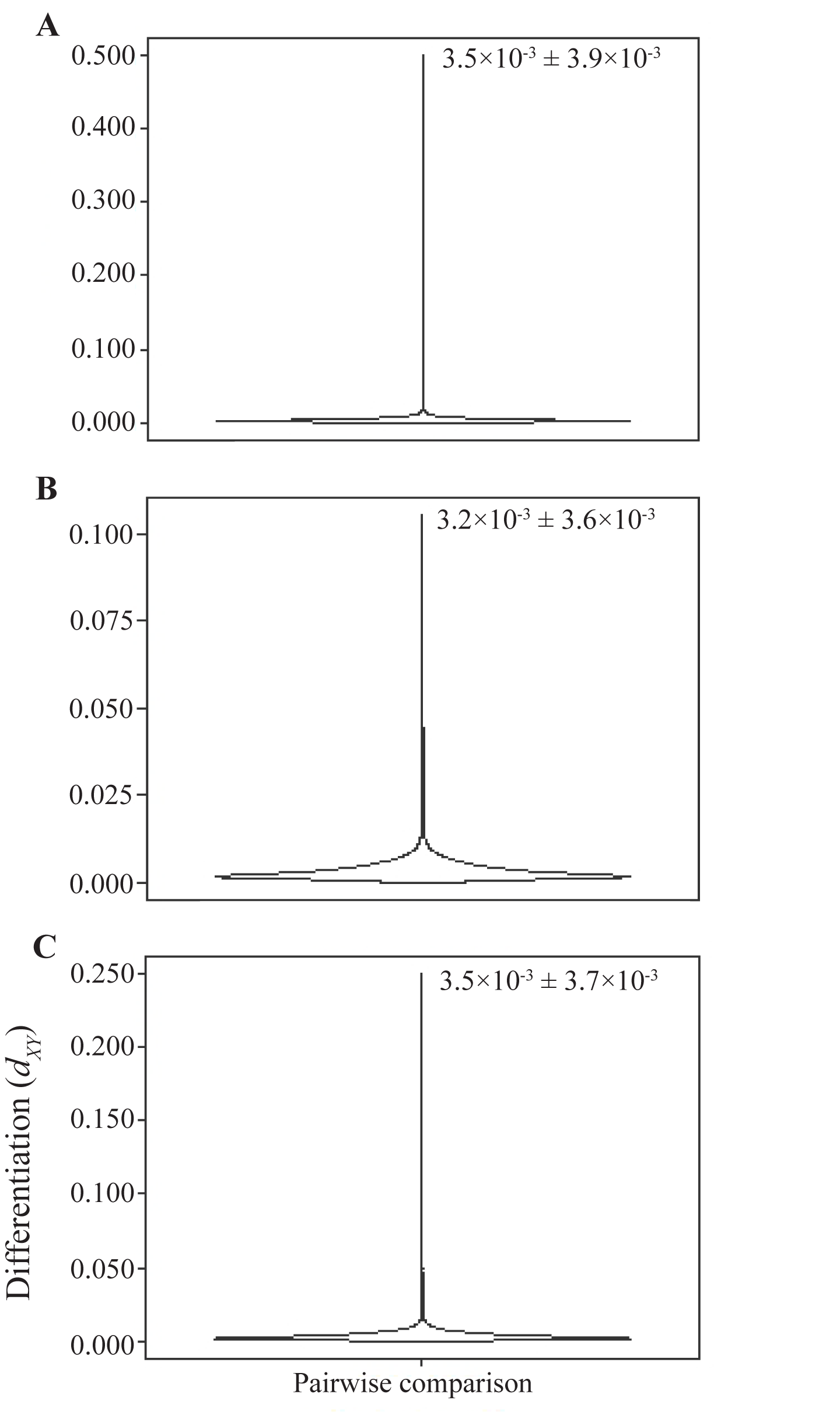

Supplement: Supplementary file 3 — Appendix S5 [file ECE3-11-1850-s003.tif]

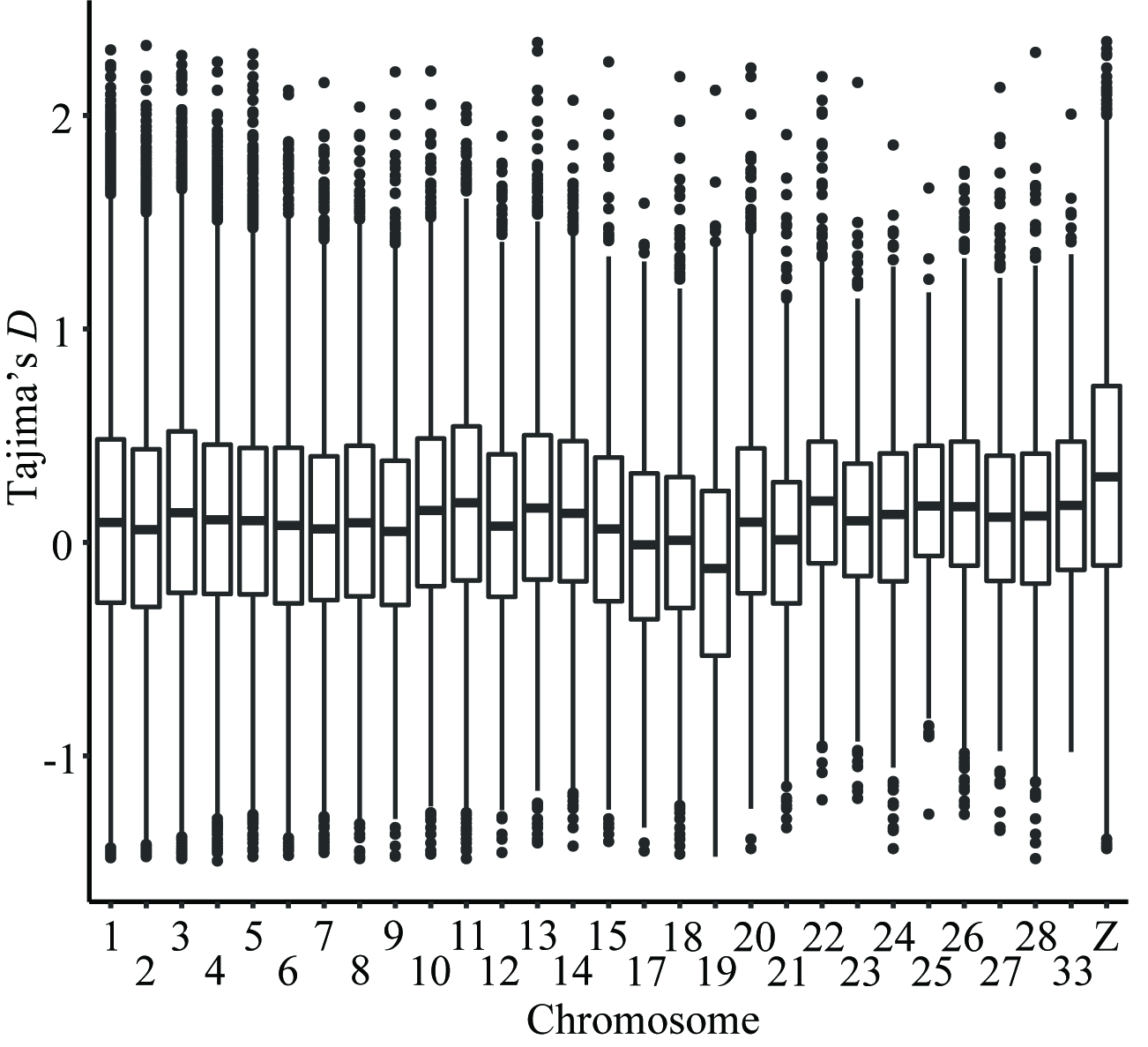

Supplement: Supplementary file 4 — Appendix S6 [file ECE3-11-1850-s004.tif]

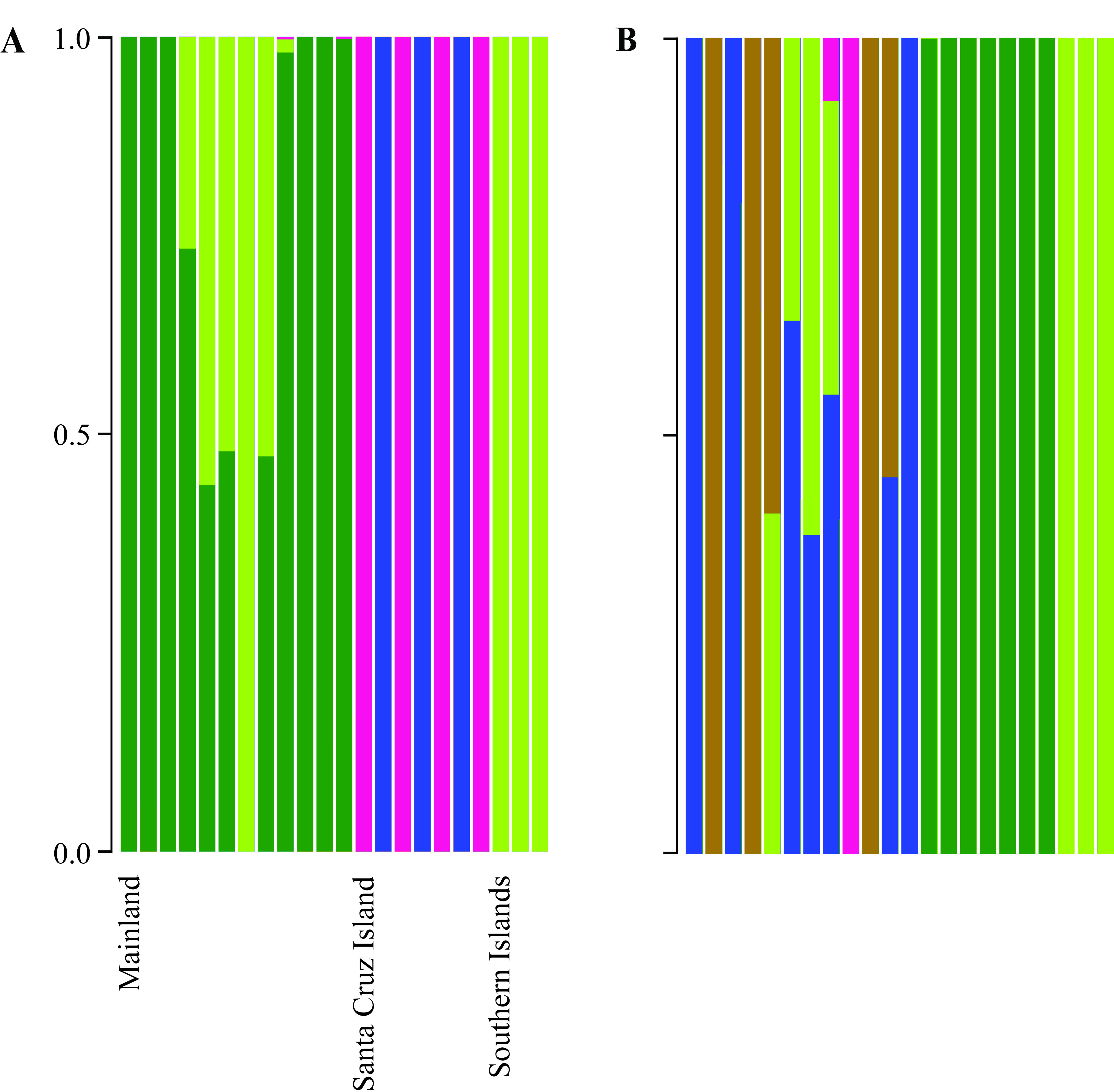

Supplement: Supplementary file 5 — Appendix S7 [file ECE3-11-1850-s005.jpg]
